# Early transcriptional responses of internalization defective *Brucella abortus* mutants in professional phagocytes, RAW 264.7

**DOI:** 10.1186/1471-2164-14-426

**Published:** 2013-06-27

**Authors:** Seung Bin Cha, Won Jung Lee, Min Kyoung Shin, Myung Hwan Jung, Seung Won Shin, An Na Yoo, Jong Wan Kim, Han Sang Yoo

**Affiliations:** 1 Department of Infectious Diseases, College of Veterinary Medicine, Brain Korea 21 for Veterinary Science, Seoul National University, Seoul 151-742, South Korea; 2 Animal and Plant Quarantine Agency, Anyang 430-016, Kyunggi, South Korea

**Keywords:** *Brucella abortus*, Infection, Macrophage, Response, Transcription

## Abstract

**Background:**

*Brucella abortus* is an intracellular zoonotic pathogen which causes undulant fever, endocarditis, arthritis and osteomyelitis in human and abortion and infertility in cattle. This bacterium is able to invade and replicate in host macrophage instead of getting removed by this defense mechanism. Therefore, understanding the interaction between virulence of the bacteria and the host cell is important to control brucellosis. Previously, we generated internalization defective mutants and analyzed the envelope proteins. The present study was undertaken to evaluate the changes in early transcriptional responses between wild type and internalization defective mutants infected mouse macrophage, RAW 264.7.

**Results:**

Both of the wild type and mutant infected macrophages showed increased expression levels in proinflammatory cytokines, chemokines, apoptosis and G-protein coupled receptors (*Gpr84*, *Gpr109a* and *Adora2b*) while the genes related with small GTPase which mediate intracellular trafficking was decreased. Moreover, cytohesin 1 interacting protein (*Cytip*) and genes related to ubiquitination (*Arrdc3* and *Fbxo21*) were down-regulated, suggesting the survival strategy of this bacterium. However, we could not detect any significant changes in the mutant infected groups compared to the wild type infected group.

**Conclusions:**

In summary, it was very difficult to clarify the alterations in host cellular transcription in response to infection with internalization defective mutants. However, we found several novel gene changes related to the GPCR system, ubiquitin-proteosome system, and growth arrest and DNA damages in response to *B. abortus* infection. These findings may contribute to a better understanding of the molecular mechanisms underlying host-pathogen interactions and need to be studied further.

## Background

*Brucella abortus* is a zoonotic pathogen that causes undulant fever, endocarditis, arthritis and osteomyelitis in humans and abortion and infertility in cattle [1,2]. They are small, non-motile, non-spore-forming Gram-negative rods and facultative intracellular organisms that are very difficult to isolate and have a long latent period that makes early diagnosis after infection impossible. Instead of producing toxins or utilizing classical virulence factors, these microbes are able to grow in phagocytes where they are inaccessible to the host humoral immune response. They also employ several intracellular survival strategies both in professional and nonprofessional phagocytic host cells [3,4]. Therefore, understanding the interaction between bacterial virulence and the host cell is important to control brucellosis.

Several studies have described the host cell responses to *Brucella* infection. Genes from macrophage RAW 264.7 demonstrated up-regulation of proinflammatory cytokines and antibacterial response-related chemokines [5,6]. In contrast, genes involved in cell cycling, apoptosis, and intracellular trafficking were decreased after four hours of *B. abortus* infection, suggesting the intracellular survival manner of this bacterium [6]. Moreover, microarray analysis of macrophages infected with three *Brucella* spp. revealed differentially expressed macrophage genes. Such studies discussed the host preference and virulence related to transcriptional responses elicited by this species [7].

In light of the available information on *B. abortus* and host interactions, we analyzed the transcriptional responses of macrophage RAW 264.7 infected with *B. abortus* mutants with defective internalization. Previously, we generated *B. abortus* mutants with defective host cellular internalization by Tn5 transposome complexes. Their envelope (CE) proteins were analyzed regarding invasion of the macrophages that resulted in the *ppk* gene and BruAb2_0168 locus, which are associated with expression of the OMP25, OMP28 and Porin2b genes, as well as pleiotropic effects of the *ccmC* gene [8]. In the present study, we infected the professional phagocyte RAW 264.7 with the *B. abortus* mutants for four hours. We then compared the early transcriptional responses of the macrophage with those of uninfected macrophages and macrophages infected with a virulent strain to evaluate the potential entry mechanism of the bacteria and host cellular responses. Possible roles in the cellular responses for the different mutants of *B. abortus* are discussed.

## Methods

### Bacterial strains and cell line

The diagnostic reference strain *Brucella abortus* 1119–3 was provided by the Animal, Plant and Fisheries Quarantine and Inspection Agency in Korea. The internalization defective mutant C10, C29, D6 and D7 were derived from our previous study [8]. *Brucellae* were cultured in Brucella broth or agar (Difco, USA), and Kanamycin (30 μg/ml) was used when necessary. RAW 264.7, a mouse leukemic monocyte macrophage cell line, was grown at 37°C in a 5% CO_2_ atmosphere in DMEM (Invitrogen, USA) containing 10% fetal bovine serum (FBS).

### Macrophage infection and RNA preparation

RAW 264.7 cells were infected with each *Brucella* strain as described previously [8]. Briefly, RAW 264.7 cells were seeded (5 × 10^6^ cells per flask) in T75 flasks one day before infection. Macrophages were infected with 1 ml of a stationary phase culture of wild type and mutant *B. abortus* strains (MOI 1,000:1). One hour post-infection, the cells were washed twice with sterile phosphate-buffered saline (PBS) and incubated with fresh media. After 4 hours of incubation, cells were washed twice with PBS, and the RNA was extracted using the RNeasy mini Kit (Qiagen, Valencia, USA) according to the manufacturer's protocol. After processing with DNase digestion and clean-up procedures, RNA samples were quantified, aliquotted, and stored at −80°C until use. For quality control, RNA purity and integrity were evaluated by denaturing the samples and performing gel electrophoresis, OD 260/280 ratio, and analyzed on the Agilent 2100 Bioanalyzer (Agilent Technologies, Palo Alto, USA). To validate the microarray results, an independent experiment was conducted with the same conditions.

### Labeling and purification

RNA amplification, labeling, array hybridization, and scanning were carried out by Macrogen Inc. (Seoul, Republic of Korea). Total RNA was amplified and purified using the Ambion Illumina RNA amplification kit (Ambion, Austin, USA) to yield biotinylated cRNA according to the manufacturer’s instructions. Briefly, 550 ng of total RNA was reverse-transcribed to cDNA using a T7 oligo(dT) primer. Second-strand cDNA was synthesized, transcribed *in vitro*, and labeled with biotin-NTP. After purification, the cRNA was quantified using the ND-1000 Spectrophotometer (NanoDrop, Wilmington, USA).

### Hybridization and data export

1.5 μg of labeled cRNA samples were hybridized to each mouse-6 expression bead array for 16–18 h at 58°C, according to the manufacturer’s instructions (Illumina, Inc., San Diego, USA). Detection of the array signal was carried out using Amersham fluorolink streptavidin-Cy3 (GE Healthcare Bio-Sciences, Little Chalfont, UK) following the bead array manual. Arrays were scanned with an Illumina bead array Reader confocal scanner according to the manufacturer’s instructions. Array data export processing and analysis were performed using Illumina BeadStudio v3.1.3 (Gene Expression Module v3.3.8).

### Raw data preparation and statistic analysis

The quality of hybridization and overall chip performance were monitored by visual inspection of both internal quality control checks and the raw scanned data. Raw data were extracted using the software provided by the manufacturer (Illumina GenomeStudio v2009.2 (Gene Expression Module v1.5.4)). Array data were filtered by detection, *p*-value < 0.05, (similar to signal to noise) in at least 50% samples. We applied a filtering criterion for data analysis; a higher signal value was required to obtain a detection *p*-value < 0.05. A selected gene signal value was transformed by logarithm and normalized by the quantile method. The comparative analysis between the test sample and control sample was carried out using fold-change.

Go-ontology analysis for a list of significant probes was performed using Protein Analysis Through Evolutionary Relationships (PANTHER) (http://www.pantherdb.org/panther/ontologies.jsp), text files containing Gene ID lists, and access numbers of illumina probe IDs. Gene Set Enrichment Analysis (GSEA) was performed to determine *a priori* if a defined set of genes showed a differential pattern for both biological processes and molecular function states. The one-tail Fisher Exact test was adopted to measure the gene-enrichment in annotation terms. All data analysis and visualization of differentially expressed genes were conducted using R 2.4.1 (http://www.r-project.org).

### Validation of microarray results

To validate the microarray results, 3 samples with increased genes, 3 samples with decreased genes, and 6 randomly selected genes (Table 1) from infected macrophages with differential expression were submitted to quantitative real time RT-PCR. Total RNA from the macrophage, the remainder used for microarray analysis, was reverse transcribed using the SuperScript® VILO™ cDNA synthesis Kit (Invitrogen, USA) according to the manufacturer’s protocol. RT-PCR reaction was performed with 1 μl of cDNA using the Rotor-Gene SYBR Green PCR kit (Qiagen) and Rotor-Gene Q real time PCR cycler (Qiagen). Amplification was done for 35 cycles at 95°C for 15 sec followed by 45 sec at 60°C with fluorescence detected during the extension phase. The expression level was determined by the 2^-ΔΔCt^ method [9] using a housekeeping gene, glyceraldehyde-3-phosphate dehydrogenase (GAPDH), as a reference. The relative expression level was compared to a respective uninfected macrophage control to determine the expression-fold change of each gene.

**Table 1:** Primers used for qRT-PCR

**Accession no.**	**Gene symbol (Description)**	**Forward primers (5′ → 3′)**	**Reverse primers (5′ → 3′)**
NM_008392.1	Irg1 (Immunoresponsive gene 1)	CCTGTGCCTCGCTGCTCGAC	CGTGTCGAAGCTTGGCGGGT
NM_007987.2	Fas (TNF receptor superfamily member 6)	CCTGCGCCCCATGCACAGAA	TCTGGGTCAGGGTGCAGTTTGT
NM_013652.2	Ccl4 (Chemokine (C-C motif) ligand 4)	GCTCTGCGTGTCTGCCCTCTC	TGGTGCTGAGAACCCTGGAGCA
NM_139154.2	Rab40c (Rab40c, member RAS oncogene family)	GACGGCGCAGCTGAATCCCC	CCAGCTTGACACGCCGTCCA
NM_028724.4	Rin2 (Ras and Rab interactor 2)	TCTGCCCTGCCTCCTTGCGT	GCACTCCAGCTCCGAAGGCG
NM_023635.5	Rab27a (Rab27a, member RAS oncogene family)	AAAAGGCCAGTCGCACGGGG	TGTCCCTGCGGTGTTGCGTC
NM_008084.2	Gapdh (Glyceraldehyde-3-phosphate dehydrogenase)	CCCCAGCAAGGACACTGAGCAAG	TGGGGGTCTGGGATGGAAATTGTG

## Results

### Microarray analysis of differentially expressed genes following infection

**Figure 1:**
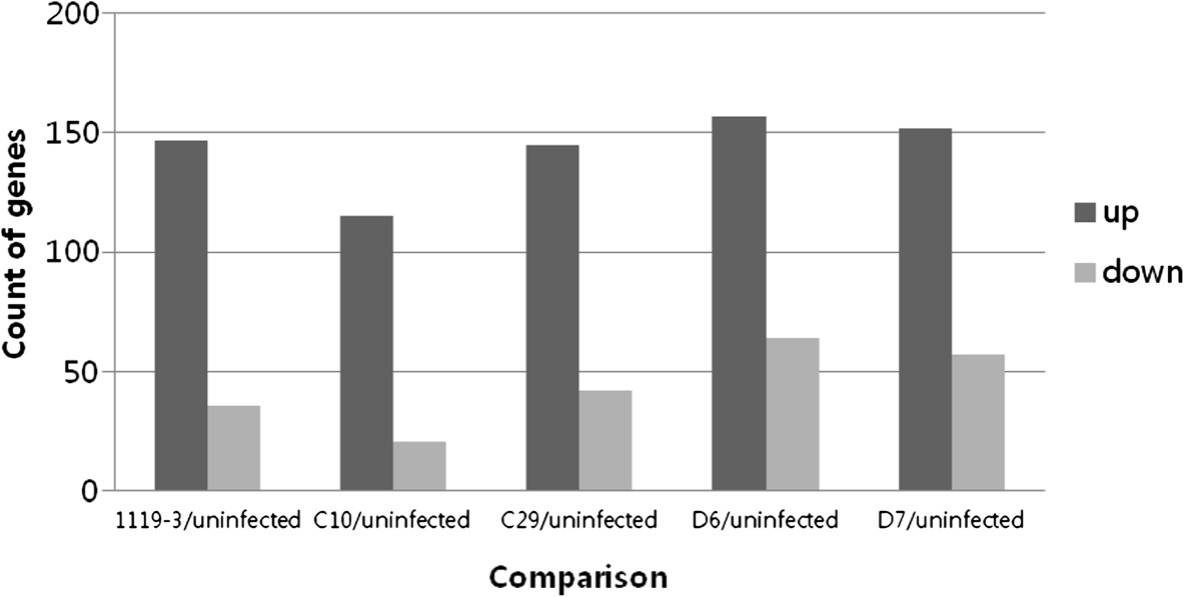
**Count of genes with up and down regulated compare to uninfected macrophage.** (*p* < 0.05, |fold change | ≥ 1.5).

This study used microarrays to analyze early transcriptional responses of murine macrophage cell line infected with wild type or mutant *B. abortus* using the Illumina Mouse WG-6 v2 Expression BeadChip which covers more than 45,000 transcripts. The threshold value for the microarray was chosen with an expression change of ≥1.5 or ≤1.5 –fold of both up- and down-regulated genes within a *p*-value of less than 0.05. Based on this criterion for selection, the 147, 115, 145, 157 and 152 genes were up-regulated and the 36, 21, 42, 64 and 57 genes were down-regulated in *B. abortus* 1119–3, C10, C29, D6 and D7 infected macrophages, respectively (Figure 1). Among the 30,854 genes analyzed, only the 183 (0.59%), 136 (0.44%), 187 (0.61%), 221 (0.72%) and 209 (0.68%) genes had altered expression levels in macrophages infected with the *B. abortus* strains 1119–3, C10, C29, D6 and D7, respectively.

The 20 most up-regulated and down-regulated genes are listed in Table 2 and 3, respectively. The *Cxcl2* gene, chemokine (C-X-C motif) ligand 2, was the most up-regulated gene with more than a 20-fold change in each experimental group. Additional genes that were strongly induced by *B. abortus* infection were related to immunity and defense (*Tnf*, *Nfkbiz*, *Ier3*, *Ccl2*, *Ccl7*, *Il1b*, *Cish* and *Nfkbia*), apoptosis (*Tnf*, *Phlda1*, *Il1b*, *Cish* and *Nfkbia*), signal transduction (*Cxcl2*, *Traf1*, *Gpr84*, *Gpr109a*, *Marcks11* and *Socs3*) and cell proliferation and differentiation (*Il1b*, *Nfkbia* and *Edn1*). These genes included *Irg1*, a previously described lipopolysaccharide (LPS)-inducible gene through a protein kinase C regulated pathway in macrophages [10].

**Table 2:** **The 20 most up-regulated genes in mouse macrophage cell line RAW 264.7 infected with each *B. abortus *compare to uninfected macrophage**

**Gene symbol**	**Description**	**1119-3**		**C10**		**C29**		**D6**		**D7**	
		**FC**	**P-value**	**FC**	**P-value**	**FC**	**P-value**	**FC**	**P-value**	**FC**	**P-value**
Cxcl2	Chemokine (C-X-C motif) ligand 2	34.68	1.56E-193	21.51	1.54E-156	33.41	7.14E-220	43.82	5.67E-166	29.62	4.21E-205
Tnf	Tumor necrosis factor	17.47	4.45E-150	14.18	3.82E-133	21.83	1.58E-168	22.45	6.39E-120	18.35	3.46E-157
Nfkbiz	Nuclear factor of kappa light polypeptide gene enhancer in B-cells inhibitor, zeta	15.58	3.68E-118	11.51	1.42E-100	18.61	3.69E-151	17.88	3.63E-96	14.34	1.80E-128
Irg1	Immunoresponsive gene 1	12.29	2.47E-99	8.82	9.26E-80	10.29	4.04E-96	14.74	1.45E-83	11.29	1.04E-106
Ier3	Immediate early response 3	8.35	4.93E-82	6.36	1.01E-63	9.03	7.44E-87	9.04	1.02E-55	7.6	2.81E-78
Traf1	NOD-derived CD11c + ve dendritic cells cDNA, RIKEN full-length enriched library, clone:F630118K07 product: Tnf receptor-associated factor 1, full insert sequence	8.13	5.31E-56	5.68	3.78E-37	7.3	1.67E-58	9.22	1.21E-56	7.54	1.42E-66
Phlda1	Pleckstrin homology-like domain, family A, member 1	6.56	3.88E-53	5.04	8.66E-43	6.97	4.86E-67	7.27	3.88E-45	6.49	9.56E-62
Gpr84	G protein-coupled receptor 84	6.30	9.74E-59	5.36	2.85E-50	6.74	1.83E-64	7..45	1.48E-45	6.48	1.31E-67
Ccl2	Chemokine (C-C motif) ligand 2	6.16	4.74E-50	4.03	1.50E-31	5.2	2.46E-48	6.16	9.30E-38	5.14	3.57E-47
Ccl7	Chemokine (C-C motif) ligand 7	5.26	8.97E-32	3.48	2.07E-18	3.68	7.03E-21	5.91	5.23E-36	4.85	1.48E-39
Il1b	Interleukin 1 beta	4.89	2.78E-26	2.87	5.56E-13	3.63	2.88E-20	6.12	1.95E-37	4.29	6.18E-33
Gpr109a	Niacin receptor 1	4.45	2.13E-23	3.31	5.81E-17	3.73	4.66E-21	5.38	3.27E-32	4.43	8.81E-35
Cish	Cytokine inducible SH2-containing protein	4.43	4.39E-23	3.0	3.85E-14	3.83	6.24E-22	5.35	5.20E-32	4.21	2.63E-32
Marcksl1	MARCKS-like 1	4.32	2.26E-29	3.29	6.83E-19	4.27	1.75E-35	4.76	1.14E-27	4.27	2.45E-35
Cd83	CD83 antigen	4.01	7.25E-26	3.31	4.48E-19	4.32	3.18E-36	4.47	1.92E-25	3.94	2.74E-31
Il4i1	Interleukin 4 induced 1	4.0	3.19E-20	3.11	4.16E-15	3.64	2.06E-20	4.56	4.58E-26	3.88	8.18E-29
Socs3	Suppressor of cytokine signaling 3	3.66	1.95E-20	2.45	3.55E-09	3.78	1.80E-26	4.93	6.06E-29	3.05	1.49E-19
Nfkbia	Nuclear factor of kappa light polypeptide gene enhancer in B-cells inhibitor, alpha	3.58	1.78E-22	3.34	2.00E-21	4.25	1.50E-36	4.46	2.12E-25	3.73	2.96E-29
Edn1	Endothelin 1	3.53	2.85E-16	2.28	8.95E-08	2.49	5.74E-10	3.51	1.20E-17	2.84	1.34E-15
Ehd1	EH-domain containing 1	3.45	8.17E-17	2.83	1.43E-12	3.22	2.12E-17	4.01	1.02E-21	3.5	1.30E-24

**Table 3:** **The 20 most down-regulated in mouse macrophage cell line RAW 264.7 infected with each *B. abortus *compare to uninfected macrophage**

**Gene symbol**	**Description**	**1119-3**		**C10**		**C29**		**D6**		**D7**	
		**FC**	**P-value**	**FC**	**P-value**	**FC**	**P-value**	**FC**	**P-value**	**FC**	**P-value**
Cytip	Cytohesin 1 interacting protein	−2.9	1.62E-11	−2.16	1.08E-06	−2.35	1.19E-08	−2.82	6.46E-12	−2.53	3.53E-13
Cxcr4	Chemokine (C-X-C motif) receptor 4	−2.17	6.43E-06	−1.81	8.36E-04	−2.13	9.94E-07	−2.63	2.59E-10	−2.12	4.46E-07
Klhl6	Kelch-like 6 (Drosophila)	−2.1	5.71E-07	−1.73	3.39E-04	−1.73	8.32E-05	−2.15	2.06E-06	−1.91	1.06E-06
Enc1	Ectodermal-neural cortex 1	−2.01	1.01E-04	−1.77	1.71E-03	−1.9	1.03E-04	−1.98	5.11E-05	−1.98	9.61E-07
Slc40a1	Solute carrier family 40 (iron-regulated transporter), member 1	−1.95	5.21E-06	−1.83	2.61E-05	−1.8	1.17E-05	−2.31	1.15E-07	−1.92	6.60E-07
Tmem86a	Transmembrane protein 86A	−1.85	1.23E-03	−1.51	>0.05	−1.79	7.58E-04	−2.02	2.43E-05	−1.79	4.44E-04
BC039093	cDNA sequence BC039093	−1.84	9.07E-04	−1.54	>0.05	−1.7	2.06E-03	−1.92	1.33E-04	−1.73	1.73E-04
5430435G22Rik	RIKEN cDNA 5430435G22 gene	−1.81	2.26E-03	−1.57	0.04	−1.74	1.90E-03	−1.93	1.25E-04	−1.68	5.34E-04
Tmem51	Transmembrane protein 51	−1.81	3.69E-04	−1.57	0.01	−1.65	8.54E-04	−2.08	9.03E-06	−1.86	4.84E-06
Lhfpl2	Lipoma HMGIC fusion partner-like 2	−1.78	1.60E-04	−1.52	0.02	−1.8	8.18E-06	−1.96	6.97E-05	−1.82	6.49E-06
Slc37a1	10 days neonate skin cDNA, RIKEN full-length enriched library, clone:4732478E01 product:solute carrier family 37 (glycerol-3-phosphate transporter), member 1, full insert sequence	−1.78	3.92E-03	−1.57	0.04	−1.61	0.02	−1.8	1.16E-03	−1.73	2.98E-04
C130050O18Rik	RIKEN cDNA C130050O18 gene	−1.78	3.88E-03	−1.62	0.02	−1.85	2.48E-04	−1.94	1.12E-04	−1.81	1.05E-04
AI595366	Leucine rich repeat containing 14B	−1.77	3.95E-03	−1.51	>0.05	−1.59	0.02	−1.85	4.90E-04	−1.63	0.01
B930041F14Rik	RIKEN cDNA B930041F14 gene	−1.75	4.00E-03	−1.54	>0.05	−1.67	3.77E-03	−1.87	3.39E-04	−1.74	1.36E-04
LOC100045981	Similar to synaptotagmin XI	−1.74	5.45E-03	−1.58	0.04	−1.65	6.70E-03	−1.97	5.60E-05	−1.85	1.29E-05
Arrdc3	Arrestin domain containing 3	−1.74	6.50E-03	−1.6	0.03	−1.71	2.72E-03	−1.95	9.29E-05	−1.55	0.02
Tspan14	Tetraspanin 14	−1.73	1.60E-03	−1.43	>0.05	−1.45	0.04	−1.72	4.53E-03	−1.52	0.01
Lzts2	Leucine zipper, putative tumor suppressor 2	−1.72	8.22E-03	−1.63	0.02	−1.74	1.82E-03	−1.87	3.22E-04	−1.76	9.06E-04
Fblim1	Filamin binding LIM protein 1	−1.72	2.29E-03	−1.53	0.04	−1.49	0.02	−1.61	0.02	−1.56	5.47E-03
Phf17	PHD finger protein 17	−1.7	0.01	−1.59	0.03	−1.62	0.01	−1.72	4.73E-03	−1.55	8.58E-03

Unlike the up-regulated genes, there were no genes with decreases of more than a 3.0 fold change. The major genes down-regulated in the mouse macrophage cell line were related to signal transduction (*Cxcr4*, *5430435G22Rik*, *Tspan14* and *Fblim1*), developmental processes (*Enc1*), cell structure and motility (*Cxcr4*, *Enc1* and *Fblim1*), nucleic acid metabolism (*BC039093* and *Phf17*) and oncogenesis (*Fblim1* and *Phf17*). Most of the top 20 down-regulated genes belonged to the biological process unclassified group (*Cytip*, *Klhl6*, *Slc40a1*, *Tmem86a*, *Tmem51*, *Lhfpl2*, *Slc37a1*, *C130050O18Rik*, *AI595366*, *B930041F14Rik*, *LOC100045981*, *Arrdc3* and *Lzts2*). However, unlike in the up-regulated genes, signal transduction was involved in intracellular protein traffic and cell adhesion mediated signaling.

### Gene enrichment and functional annotation analysis

To detect coordinated changes in pre-specified sets of related genes, gene enrichment and functional annotation were analyzed. Differentially expressed genes were categorized by biological process and molecular function state using the PANTHER classification database by means of Fisher’s exact test.

**Figure 2:**
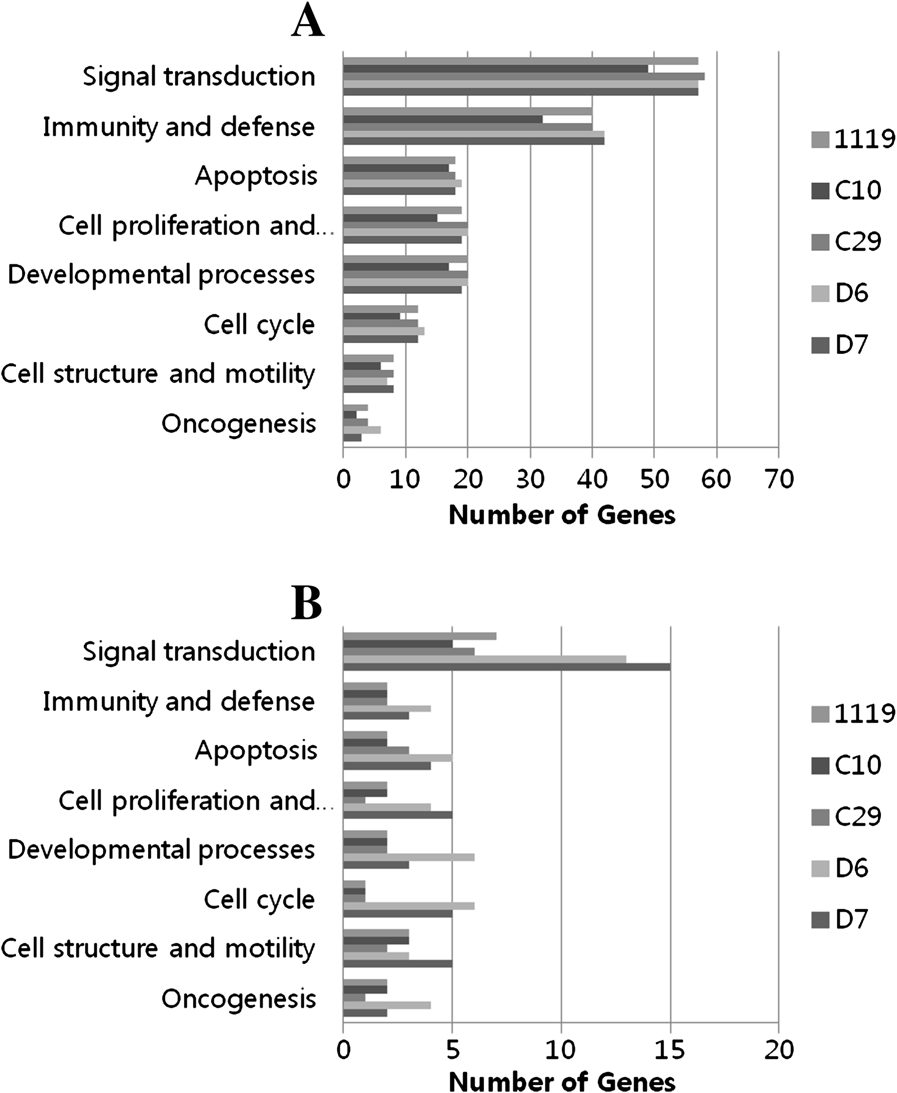
**Categorization by biological process of genes with significantly regulated transcripts in RAW 264.7 macrophage at 4 h post infection of each *B. abortus *strains.** Up-regulated transcripts (**A**) and down-regulated transcripts (**B**). (*P* < 0.05, fisher’s exact test).

**Figure 3:**
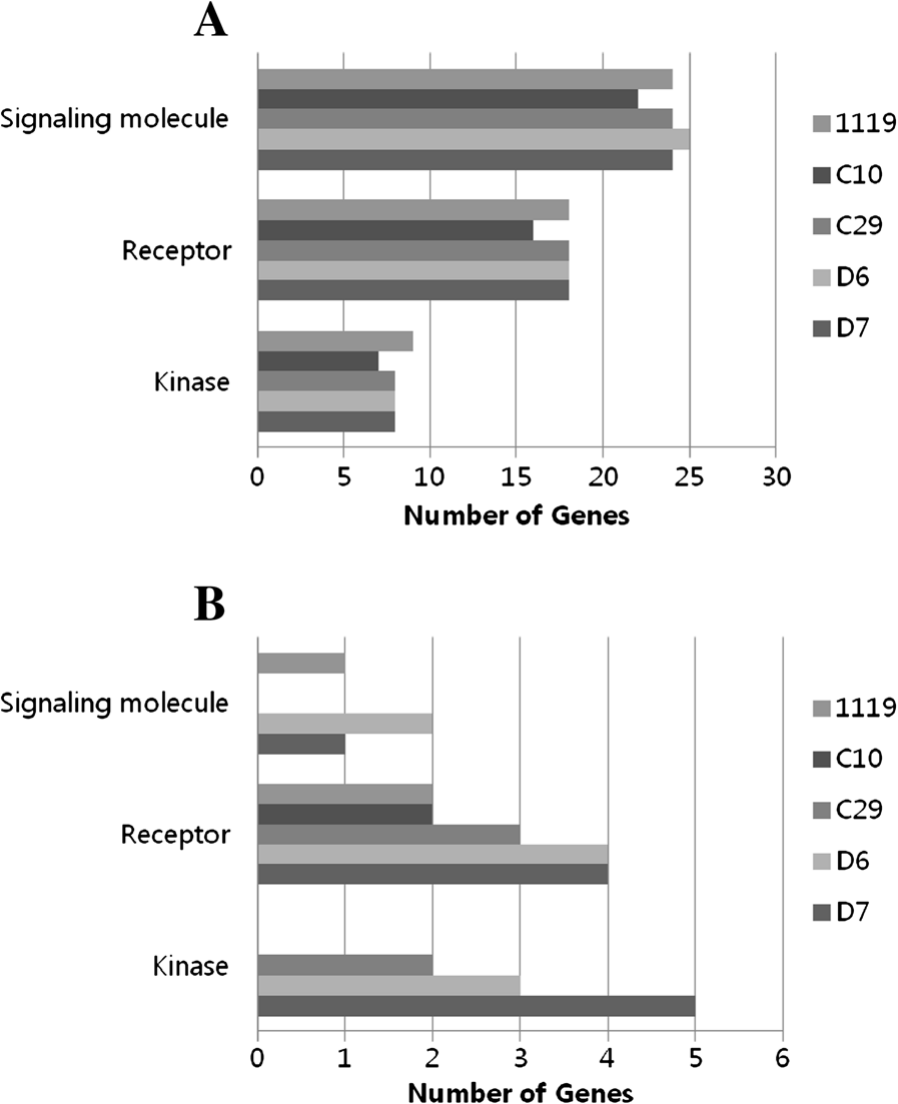
**Categorization by molecular function of genes with significantly regulated transcripts in RAW 264.7 macrophage at 4 h post infection of each *B. abortus *strains.** Up-regulated transcripts (**A**) and down-regulated transcripts (**B**). (*P* < 0.05, fisher’s exact test).

As a result, 8 biological process categories (signal transduction, immunity and defense, apoptosis, cell proliferation and differentiation, developmental process, cell cycle, cell structure and motility and oncogenesis) and 3 molecular function categories (signaling molecule, receptor and kinase) were found to be associated with early transcriptional changes following *B. abortus* infection (Figure 2 and 3). A full list of genes with altered expression levels is provided in Additional files 1 and 2.

Most of the up-regulated genes involved in the signal transduction category were cytokines (*Tnf*, *Il1a*, *Il1b, Ltb* and *Csf2*) and chemokines (*Cxcl2*, *Ccl2*, *Ccl7*, *Ccl3* and *Ccl9*), which are also related to host immune response and defense, similar to the previously reported transcriptional analysis of *B. melitensis* infected macrophages [7]. Like the proinflammatory cytokines and chemokines, genes involved in the apoptosis category (*Fas*, *Traf1* and *Ripk2*) showed an increased transcription level as a response to an intracellular pathogen. This is a useful way for the host to eliminate infected cells, decreasing the likelihood of spread of the infection to neighboring cells and preventing pathogenicity. However, several genes involved in the inhibition of apoptosis (*Cish* and *Socs3*) were up-regulated as an effort to enhance bacterial survival in the host cell. In the signal transduction category, *Gpr84*, *Gpr109a* and *Adora2b* genes related to G-protein coupled receptors (GPCRs) were up-regulated following *B. abortus* infection.

In contrast to the up-regulated genes, genes down-regulated in the signal transduction category were *Rab40c*, *Rin2* and *5430435G22Rik*, small GTPases which mediate intracellular trafficking of this bacterium without affecting internalization [11]. These were also down-regulated in macrophages infected with other *Brucella* spp. [7]. Although more than 50% of the down-regulated genes were categorized as unclassified, cytohesin 1 interacting protein (*Cytip*), a membrane-bounded organelle that carries materials newly ingested by endocytosis and passes many of the materials to lysosomes for degradation, was down-regulated. In addition, genes related to ubiquitination were down-regulated (*Arrdc3* and *Fbxo21*), suggesting the survival strategy of this bacterium.

### Differentially expressed genes in internalization defective mutant infected cells compare to wild type infected cell

**Figure 4:**
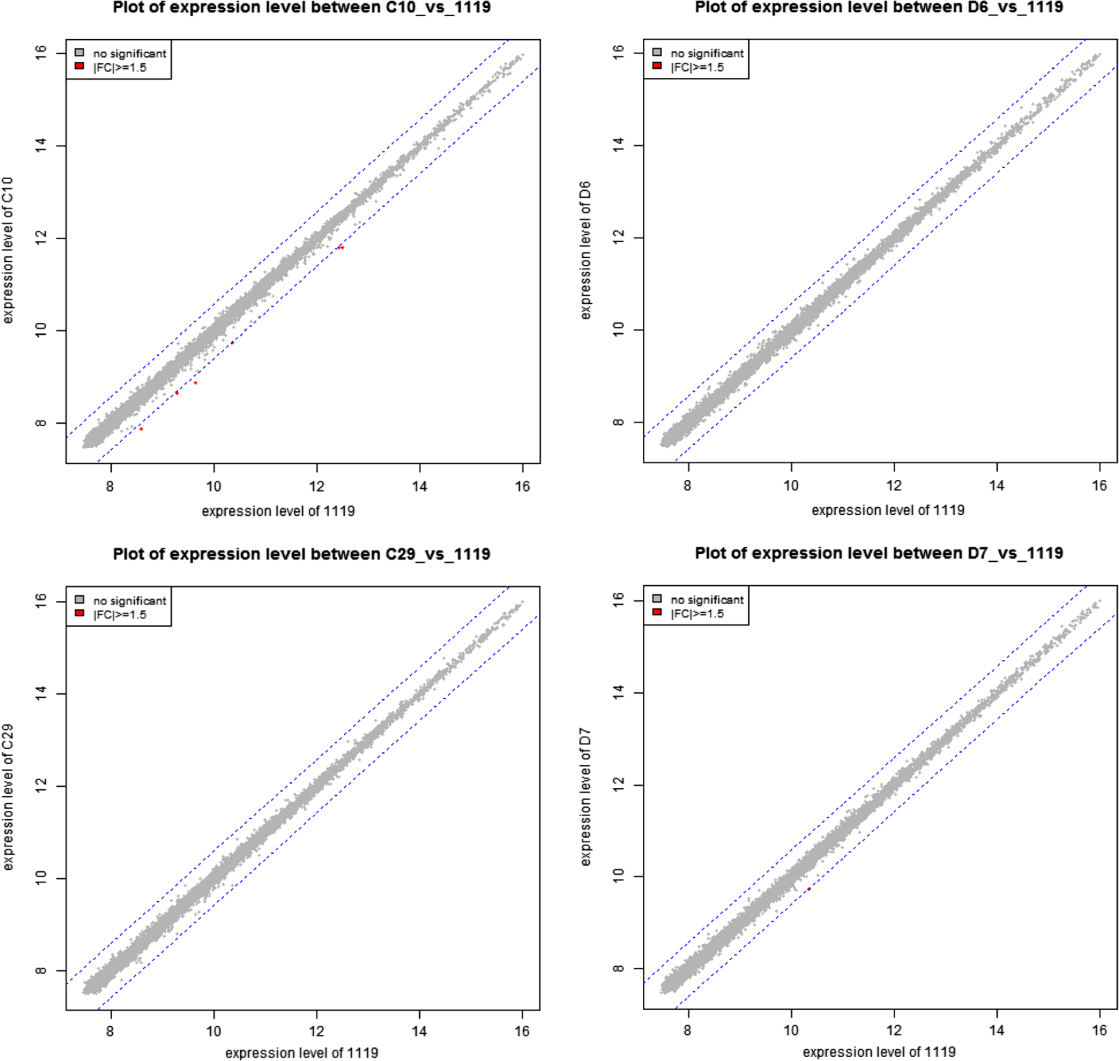
**Plots of the expression level between wild type infected cell versus mutant infected cell.** Red dots indicate an expression level change of ≥1.5 or ≤1.5 fold of both up- and down- regulated genes. Expression level was calculated by base 2 logarithm of normalized hybridization signals from each sample.

To analyze the genes with altered transcription between wild type and mutant infected macrophages, we plotted the median of the normalized hybridization signals of the wild type infected cells against the cells infected with the four other mutants (Figure 4). As shown in the graphs, most of the genes in each mutant infected group lie within a diagonal where expression is equivalent between the groups, indicating that the majority of genes are expressed at similar levels when compared to the wild type infected group. There was no up-regulated gene in the mutant infected groups compared to the wild type infected group. There were 6 (*Il1b*, *Lcn2*, *Cxcl2*, *Edn1*, *Ccl2* and *Ccl7*) and 1 (*Rn18s*) down-regulated genes in the groups infected with mutants C10 and D7 compared to the wild type infected group, respectively. However, only two genes, *Cxcl2* (Chemokine C-X-C motif ligand 2) and *Ccl2* (Chemokine C-C motif ligand 2) in the C10 infected group were statistically significant (*P* < 0.05). These genes are chemotactic for polymorphonuclear leukocytes and monocytes to the sites of infection.

### Validation of microarray data

**Figure 5:**
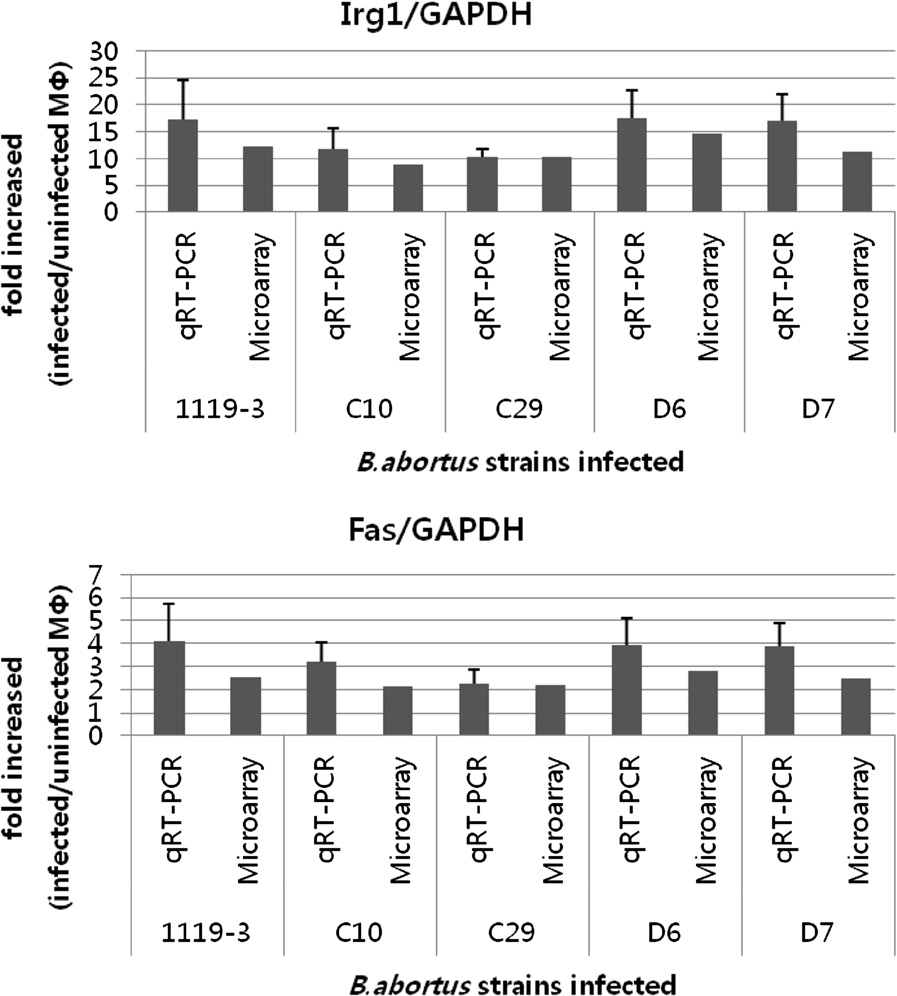
**Validation of microarray data by quantitative RT-PCR.** The relative expression level was normalized by *Gapdh* expression level and relative to uninfected cells. RT-PCR data were averaged from three independent RNA isolation and presented as mean relative expression with an error bar which represents SEM.

To confirm the microarray data, we performed quantitative RT-PCR with randomly selected genes. We selected *Irg1*, *Fas* and *Ccl4* genes from the up-regulated gene group and *Rab40c*, *Rin2* and *Rab27a* genes from the down-regulated gene group. We could validate the microarray data because all genes tested by qRT-PCR showed more fluctuation (increased or decreased) than the microarray data, but in the same direction (Figure 5). We also attempted to analyze the significant changes in mutant infected cells compared to wild type infected cells with qRT-PCR data from *Rab40c*, *Rin2* and *Rab27a*, which showed both more or less than 1.5-fold changes according to the infected strains with microarray data. However, we could not detect any significant changes in the mutant infected groups compared to the wild type infected group, as shown in the microarray analysis data.

## Discussion

Understanding the host-pathogen interaction is very important to reveal the mechanisms of the pathogen related to global host gene regulation during infection, genomics, and mechanisms of secretion of bacterial virulence factors, especially for intracellular bacteria. Microarray is a powerful tool that can increase our knowledge about the host-pathogen interaction by investigating host responses to the pathogen infection and vice versa [12,13]. *B. abortus* is an intracellular pathogen that can survive and replicate within host macrophages. In light of this, to investigate the host-pathogen interaction of our internalization defective *B. abortus* mutants described previously [8], we performed microarray analysis with 30,854 murine genes following *B. abortus* infection of the RAW 264.7 macrophage. As four hours of infection was enough to elicit specific transcriptional responses in macrophages infected with different *Brucella* spp. [7], we also analyzed the macrophages following four hours of infection.

The overall transcriptional profile was similar to the previous study of infected *B. abortus* strain 2308 [6], although we used different microarray chips covering more than 30,000 genes. However, we found that some genes involved with the G protein coupled receptor (GPCR) showed an increased expression level compared to the non-infected group. The G protein-coupled receptor 84 (GPR84) is induced in monocytes and macrophages and functions as a specific receptor for medium-chain free fatty acids (FFAs) of C_9_ to C_14_ length. It also amplifies LPS-stimulated IL-12 p40 production and is coupled to a pertussis toxin-sensitive G_i/o_ pathway once activated [14]. The pertussis toxin is secreted by the pertussis toxin liberation (Ptl) type IV secretion system (T4SS) of *Bordetella pertussis*[15] and results in accumulation of intracellular cyclic adenosine monophosphate (cAMP) [16]. This result suggests that the virB type IV secretion system (T4SS) of *B. abortus*, which is core virulence factor of this bacterium [17] as well as a mediator for host innate immune response [18], might secret some effector molecules that acts to increase intracellular cAMP for intracellular survival [19] via GPCR of the host cell. The other GPCR, GPR109A, is a member of the nicotinic acid receptor family of GPCRs that reduces the level of intracellular cAMP following inhibition of lipolysis in adipocytes [20]. Moreover, the effects of nicotinic acid on macrophages, spleen and probably adipocytes are mediated via an identical, unique G protein-coupled receptor [21]. This suggests that *B. abortus* may utilize the GPCR system to prevent lipolytic processing within phagosomes in spite of cAMP reduction. As the previous study showed, the regulator of G protein signaling 2 (RGS2) expression was induced following *B. abortus* infection [22]. We also found several regulators of G protein signaling (*Arhgef3*, *Rassf4* and *Rgs16*) with increased expression levels, although the precise mechanism remains to be elucidated. Taken together, these alterations in the G protein mediated signaling system may result in increased survival of *B. abortus* within the macrophage.

Interestingly, *Cxcr4*, a gene coding chemokine (C-X-C motif) receptor 4 (CXCR4), was down-regulated, whereas other chemokine-mediated genes had been up-regulated. As the CXCR4 expression is reduced by inflammatory cytokines such as tumor necrosis factor-α (TNF- α) and interleukin-1β (IL-1β) [23], we considered this a consequence of up-regulation of *Tnf* and *Il1b*. However, a recent study showed that extracellular ubiquitin is a natural ligand of CXCR4 [24], and we also found up-regulated (*Znrf1*, *Herpud1* and *Socs3*) and down-regulated (*Cxcr4*, *Fbxo21* and *Rab40c*) genes involved in the ubiquitin-proteasome system. Although CXCR4 is a member of GPCR, which could affect the other signaling cascades, and a receptor for extracellular ubiquitin, it has been shown that cellular uptake of extracellular ubiquitin results in its covalent conjugation to intracellular proteins of the target cell [23,25]. In light of this, the exact function of remaining genes is not fully understood, yet we may speculate that the host cell utilizes the ubiquitin-proteasome system in an effort to clear this pathogen and controlling this system is a bacterial survival strategy.

*Gadd45* is a growth arrest and DNA damage gene and includes *Gadd45a*, *Gadd45b* and *Gadd45g*. A previous study found that *Gadd45a* was induced in response to DNA damage and function to inhibit the growth of damaged cells in *Brucella* infected macrophages [6,7]. In addition, increased expression of *Gadd45b* was observed, indicating the regulatory roles of activated macrophages against *Brucella* infection [7] as well as anti-apoptotic activity since *Gadd45a-* and *Gadd45b-* deficient mice were sensitized to genotoxic-stress-induced apoptosis [26]. In this study, we also observed increased expression of both *Gadd45a* and *Gadd45b*; however, we found the expression level of *Gadd45g* gene was decreased. Gadd45a, Gadd45b and Gadd45g serve similar, but not identical, functions along different apoptotic and growth inhibitory pathways [27,28] and Gadd45g acts as a positive mediator of apoptosis in response to genetic and environmental stress [29,30]. This suggests *Gadd45g* was down-regulated to protect against apoptosis, though the outcomes of Gadd45 function are determined by the stress stimulus encountered, cell type, and interactions with other proteins [26].

In spite of these novel genes identified with altered expression levels compared to uninfected macrophages, we could not detect any genes that changed in a different direction. Only two genes (*Cxcl2* and *Ccl2*) in the mutant C10 infected group were slightly decreased in the same direction as wild type infected macrophages. As our mutants did not show a fully defective internalization phenotype or complete deletions in bacterial cellular envelope components [8], we assumed that a very small amount of bacteria could elicit a response in the host cell. However, considering both *Salmonella typhimurium* infected macrophages and purified LPS inducted macrophages showed similar changes in gene expression [31] and all of mutants used in this study were smooth strains, we concluded that an internalization deficiency in *B. abortus* would not affect transcriptional changes in macrophages if there was LPS contained. This is consistent with a previous study that showed few transcriptional changes in macrophages infected with different *Brucella* species including both smooth (*B. melitensis* and *B. neotomae*) and rough LPS strains (*B.ovis*) [7].

## Conclusions

In summary, it was very difficult to clarify the alterations in host cellular transcription in response to infection with internalization defective mutants. Thus, we concluded mutations within the *ccmC*, *ppk*, BruAb1_1377 and BruAb2_0168 loci would not affect the host cellular responses. However, we found several novel gene changes related to the GPCR system, ubiquitin-proteosome system, and growth arrest and DNA damages in response to *B. abortus* infection. We thus speculated about the virulence factors of this bacterium, including T4SS and its translocation of potential substrates. These findings may contribute to a better understanding of the molecular mechanisms underlying host-pathogen interactions and need to be studied further.

## Competing interests

The authors declare that they have no competing interests.

## Authors’ contributions

SBC performed overall experiments, analyzed data and wrote the manuscript. WJL, MKS and MHJ coordinated in generation of mutant strains. SWS and ANY coordinated in qRT-PCR and statistical analyses. JWK and HSY provided guidance and helped coordination. All authors read and approved the final manuscript.

## Supplementary Material

Additional file 1 **Genes with up-regulated in RAW 264.7 infected with each *B.abortus *compare to uninfected macrophage.** Click here for file

Additional file 2 **Genes with down-regulated in RAW 264.7 infected with each *B.abortus *compare to uninfected macrophage.** Click here for file

## References

[B1] Boschiroli ML Foulongne V O'Callaghan D Brucellosis: a worldwide zoonosis Curr Opin Microbiol 2001 4 1 58 64 11173035 10.1016/s1369-5274(00)00165-x

[B2] Franco MP Mulder M Gilman RH Smits HL Human brucellosis Lancet Infect Dis 2007 7 12 775 786 18045560 10.1016/S1473-3099(07)70286-4

[B3] Pizarro-Cerdá J Moreno E Sanguedolce V Mege JL Gorvel JP Virulent Brucella abortus prevents lysosome fusion and is distributed within autophagosome-like compartments Infect Immun 1998 66 5 2387 2392 9573138 10.1128/iai.66.5.2387-2392.1998 PMC108212

[B4] Rittig MG Alvarez-Martinez MT Porte F Liautard JP Rouot B Intracellular survival of Brucella spp. in human monocytes involves conventional uptake but special phagosomes Infect Immun 2001 69 6 3995 4006 11349069 10.1128/IAI.69.6.3995-4006.2001 PMC98462

[B5] Oliveira SC Harms JS Rech EL Rodarte RS Bocca AL Goes AM Splitter GA The role of T-cell subsets and cytokines in the regulation of intracellular bacterial infection Braz J Med Biol Res 1998 31 1 77 84 9686182 10.1590/s0100-879x1998000100010

[B6] Eskra L Mathison A Splitter G Microarray analysis of mRNA levels from RAW 264.7 macrophages infected with Brucella abortus Infect Immun 2003 71 3 1125 1133 12595423 10.1128/IAI.71.3.1125-1133.2003 PMC148819

[B7] Covert J Mathison AJ Eskra L Banai M Splitter G Brucella melitensis, B. neotomae and B. ovis elicit common and distinctive macrophage defense transcriptional responses Exp Biol Med (Maywood) 2009 234 12 1450 1467 19934366 10.3181/0904-RM-124 PMC2880867

[B8] Cha SB Rayamajhi N Lee WJ Shin MK Jung MH Shin SW Kim JW Yoo HS Generation and envelope protein analysis of internalization defective Brucella abortus mutants in professional phagocytes, RAW 264.7 FEMS Immunol Med Microbiol 2012 64 2 244 254 22066675 10.1111/j.1574-695X.2011.00896.x

[B9] Livak KJ Schmittgen TD Analysis of relative gene expression data using real-time quantitative PCR and the 2(−Delta Delta C(T)) Method Methods 2001 25 4 402 408 11846609 10.1006/meth.2001.1262

[B10] Chen B Zhang D Pollard JW Progesterone regulation of the mammalian ortholog of methylcitrate dehydratase (immune response gene 1) in the uterine epithelium during implantation through the protein kinase C pathway Mol Endocrinol 2003 17 11 2340 2354 12893884 10.1210/me.2003-0207

[B11] Chaves-Olarte E Guzmán-Verri C Méresse S Desjardins M Pizarro-Cerdá J Badilla J Gorvel JP Moreno E Activation of Rho and Rab GTPases dissociates Brucella abortus internalization from intracellular trafficking Cell Microbiol 2002 4 10 663 676 12366403 10.1046/j.1462-5822.2002.00221.x

[B12] Leroy Q Raoult D Review of microarray studies for host-intracellular pathogen interactions J Microbiol Methods 2010 81 2 81 95 20188126 10.1016/j.mimet.2010.02.008

[B13] Kato-Maeda M Gao Q Small PM Microarray analysis of pathogens and their interaction with hosts Cell Microbiol 2001 3 11 713 719 11696031 10.1046/j.1462-5822.2001.00152.x

[B14] Wang J Wu X Simonavicius N Tian H Ling L Medium-chain fatty acids as ligands for orphan G protein-coupled receptor GPR84 J Biol Chem 2006 281 45 34457 34464 16966319 10.1074/jbc.M608019200

[B15] Backert S Meyer TF Type IV secretion systems and their effectors in bacterial pathogenesis Curr Opin Microbiol 2006 9 2 207 217 16529981 10.1016/j.mib.2006.02.008

[B16] Carbonetti NH Pertussis toxin and adenylate cyclase toxin: key virulence factors of Bordetella pertussis and cell biology tools Future Microbiol 2010 5 3 455 469 20210554 10.2217/fmb.09.133 PMC2851156

[B17] Celli J de Chastellier C Franchini DM Pizarro-Cerda J Moreno E Gorvel JP Brucella evades macrophage killing via VirB-dependent sustained interactions with the endoplasmic reticulum J Exp Med 2003 198 4 545 556 12925673 10.1084/jem.20030088 PMC2194179

[B18] Roux CM Rolán HG Santos RL Beremand PD Thomas TL Adams LG Tsolis RM Brucella requires a functional Type IV secretion system to elicit innate immune responses in mice Cell Microbiol 2007 9 7 1851 1869 17441987 10.1111/j.1462-5822.2007.00922.x

[B19] de Bagues MP J Dudal S Dornand J Gross A Cellular bioterrorism: how Brucella corrupts macrophage physiology to promote invasion and proliferation Clin Immunol 2005 114 3 227 238 15721833 10.1016/j.clim.2004.07.010

[B20] Zhang Y Schmidt RJ Foxworthy P Emkey R Oler JK Large TH Wang H Su EW Mosior MK Eacho PI Cao G Niacin mediates lipolysis in adipose tissue through its G-protein coupled receptor HM74A Biochem Biophys Res Commun 2005 334 2 729 732 16018973 10.1016/j.bbrc.2005.06.141

[B21] Lorenzen A Stannek C Burmeister A Kalvinsh I Schwabe U G protein-coupled receptor for nicotinic acid in mouse macrophages Biochem Pharmacol 2002 64 4 645 648 12167483 10.1016/s0006-2952(02)01220-0

[B22] Kim DH Lim JJ Lee JJ Kim DG Lee HJ Min W Kim KD Chang HH Endale M Rhee MH Watarai M Kim S RGS2-mediated intracellular Ca2+ level plays a key role in the intracellular replication of Brucella abortus within phagocytes J Infect Dis 2012 205 3 445 452 22158566 10.1093/infdis/jir765

[B23] Gupta SK Lysko PG Pillarisetti K Ohlstein E Stadel JM Chemokine receptors in human endothelial cells. Functional expression of CXCR4 and its transcriptional regulation by inflammatory cytokines J Biol Chem 1998 273 7 4282 4287 9461627 10.1074/jbc.273.7.4282

[B24] Saini V Marchese A Majetschak M CXC chemokine receptor 4 is a cell surface receptor for extracellular ubiquitin J Biol Chem 2010 285 20 15566 15576 20228059 10.1074/jbc.M110.103408 PMC2865327

[B25] Daino H Matsumura I Takada K Odajima J Tanaka H Ueda S Shibayama H Ikeda H Hibi M Machii T Hirano T Kanakura Y Induction of apoptosis by extracellular ubiquitin in human hematopoietic cells: possible involvement of STAT3 degradation by proteasome pathway in interleukin 6-dependent hematopoietic cells Blood 2000 95 8 2577 2585 10753837

[B26] Gupta M Gupta SK Balliet AG Hollander MC Fornace AJ Hoffman B Liebermann DA Hematopoietic cells from Gadd45a- and Gadd45b-deficient mice are sensitized to genotoxic-stress-induced apoptosis Oncogene 2005 24 48 7170 7179 16170381 10.1038/sj.onc.1208847

[B27] Selvakumaran M Lin HK Sjin RT Reed JC Liebermann DA Hoffman B The novel primary response gene MyD118 and the proto-oncogenes myb, myc, and bcl-2 modulate transforming growth factor beta 1-induced apoptosis of myeloid leukemia cells Mol Cell Biol 1994 14 4 2352 2360 8139540 10.1128/mcb.14.4.2352-2360.1994 PMC358602

[B28] Vairapandi M Balliet AG Hoffman B Liebermann DA GADD45b and GADD45g are cdc2/cyclinB1 kinase inhibitors with a role in S and G2/M cell cycle checkpoints induced by genotoxic stress J Cell Physiol 2002 192 3 327 338 12124778 10.1002/jcp.10140

[B29] Wang H Yadav JS Global gene expression changes underlying Stachybotrys chartarum toxin-induced apoptosis in murine alveolar macrophages: evidence of multiple signal transduction pathways Apoptosis 2007 12 3 535 548 17186382 10.1007/s10495-006-0008-x

[B30] Zerbini LF Czibere A Wang Y Correa RG Otu H Joseph M Takayasu Y Silver M Gu X Ruchusatsawat K Li L Sarkar D Zhou JR Fisher PB Libermann TA A novel pathway involving melanoma differentiation associated gene-7/interleukin-24 mediates nonsteroidal anti-inflammatory drug-induced apoptosis and growth arrest of cancer cells Cancer Res 2006 66 24 11922 11931 17178890 10.1158/0008-5472.CAN-06-2068

[B31] Rosenberger CM Scott MG Gold MR Hancock RE Finlay BB Salmonella typhimurium infection and lipopolysaccharide stimulation induce similar changes in macrophage gene expression J Immunol 2000 164 11 5894 5904 10820271 10.4049/jimmunol.164.11.5894

