# Supplementary material for: Early transcriptional responses of internalization defective Brucella abortus mutants in professional phagocytes, RAW 264.7
Source: BMC Genomics. 2013 Jun 27;14:426. doi: 10.1186/1471-2164-14-426 (PMC3716731; doi:10.1186/1471-2164-14-426)
Supplement: Additional file 1 — Genes with up-regulated in RAW 264.7 infected with each B.abortus compare to uninfected macrophage. [file 1471-2164-14-426-S1.docx]

Additional file 1. Genes with up-regulated in RAW 264.7 infected with each *B.abortus* compare to uninfected macrophage.

| Gene symbol | | Description | 1119-3 | | C10 | | C29 | | D6 | | D7 | |
| --- | --- | --- | --- | --- | --- | --- | --- | --- | --- | --- | --- | --- |
|  |  |  | FC | P-value | FC | P-value | FC | P-value | FC | P-value | FC | P-value |
| Signal transduction | | |  |  |  |  |  |  |  |  |  |  |
| Cxcl2 | Chemokine (C-X-C motif) ligand 2 | | 34.68 | 1.56E-193 | 21.51 | 1.54E-156 | 33.41 | 7.14E-220 | 43.82 | 5.67E-166 | 29.62 | 4.21E-205 |
| Tnf | Tumor necrosis factor | | 17.47 | 4.45E-150 | 14.18 | 3.82E-133 | 21.83 | 1.58E-168 | 22.45 | 6.39E-120 | 18.35 | 3.46E-157 |
| Gpr84 | G protein-coupled receptor 84 | | 6.3 | 9.74E-59 | 5.36 | 2.85E-50 | 6.74 | 1.83E-64 | 7.45 | 1.48E-45 | 6.48 | 1.31E-67 |
| Ccl2 | Chemokine (C-C motif) ligand 2 | | 6.16 | 4.74E-50 | 4.03 | 1.50E-31 | 5.2 | 2.46E-48 | 6.16 | 9.30E-38 | 5.14 | 3.57E-47 |
| Ccl7 | Chemokine (C-C motif) ligand 7 | | 5.26 | 8.97E-32 | 3.48 | 2.07E-18 | 3.68 | 7.03E-21 | 5.91 | 5.23E-36 | 4.85 | 1.48E-39 |
| Il1b | Interleukin 1 beta | | 4.89 | 2.78E-26 | 2.87 | 5.56E-13 | 3.63 | 2.88E-20 | 6.12 | 1.95E-37 | 4.29 | 6.18E-33 |
| Gpr109a | Niacin receptor 1 | | 4.45 | 2.13E-23 | 3.31 | 5.81E-17 | 3.73 | 4.66E-21 | 5.38 | 3.27E-32 | 4.43 | 8.81E-35 |
| Cish | Cytokine inducible SH2-containing protein | | 4.43 | 4.39E-23 | 3.0 | 3.85E-14 | 3.83 | 6.24E-22 | 5.35 | 5.20E-32 | 4.21 | 2.63E-32 |
| Marcksl1 | MARCKS-like 1 | | 4.32 | 2.26E-29 | 3.29 | 6.83E-19 | 4.27 | 1.75E-35 | 4.76 | 1.14E-27 | 4.27 | 2.45E-35 |
| Socs3 | Suppressor of cytokine signaling 3 | | 3.66 | 1.95E-20 | 2.45 | 3.55E-09 | 3.78 | 1.80E-26 | 4.93 | 6.06E-29 | 3.05 | 1.49E-19 |
| Nfkbia | Nuclear factor of kappa light polypeptide gene enhancer in B-cells inhibitor, alpha | | 3.58 | 1.78E-22 | 3.34 | 2.00E-21 | 4.25 | 1.50E-36 | 4.46 | 2.12E-25 | 3.73 | 2.96E-29 |
| Edn1 | Endothelin 1 | | 3.53 | 2.85E-16 | 2.28 | 8.95E-08 | 2.49 | 5.74E-10 | 3.51 | 1.20E-17 | 2.84 | 1.34E-15 |
| LOC240672 | Similar to MAP-kinase phosphatase (cpg21) | | 3.15 | 5.43E-16 | 2.8 | 4.99E-13 | 3.66 | 9.59E-27 | 3.58 | 2.96E-18 | 3.37 | 7.41E-24 |
| Ccrl2 | Chemokine (C-C motif) receptor-like 2 | | 2.93 | 1.09E-11 | 2.41 | 6.64E-09 | 2.87 | 2.58E-13 | 3.61 | 1.73E-18 | 2.74 | 2.19E-15 |
| Vegfc | Vascular endothelial growth factor C | | 2.77 | 1.05E-10 | 2.36 | 2.38E-08 | 2.27 | 5.94E-08 | 3.03 | 1.18E-13 | 2.63 | 2.16E-14 |
| Cd40 | CD40 antigen, transcript variant 5 | | 2.73 | 3.72E-10 | 2.13 | 1.99E-06 | 2.23 | 1.37E-07 | 2.97 | 3.40E-13 | 2.45 | 1.60E-11 |
| Gene symbol | | Description | 1119-3 | | C10 | | C29 | | D6 | | D7 | |
|  |  |  | FC | P-value | FC | P-value | FC | P-value | FC | P-value | FC | P-value |
| Signal transduction (*Continued*) | | |  |  |  |  |  |  |  |  |  |  |
| Icam1 | Intercellular adhesion molecule 1 | | 2.54 | 1.15E-12 | 2.52 | 1.27E-13 | 2.76 | 1.25E-17 | 3.0 | 1.94E-13 | 2.76 | 5.20E-18 |
| Il13ra2 | Interleukin 13 receptor, alpha 2 | | 2.53 | 1.08E-08 | 2.01 | 1.91E-05 | 2.29 | 3.80E-08 | 2.81 | 8.06E-12 | 2.49 | 1.74E-11 |
| Fas | Fas (TNF receptor superfamily member 6) | | 2.51 | 1.55E-08 | 2.11 | 3.06E-06 | 2.21 | 2.21E-07 | 2.82 | 7.05E-12 | 2.47 | 5.30E-12 |
| Tnfrsf1b | Tumor necrosis factor receptor superfamily, member 1b | | 2.51 | 1.57E-08 | 1.92 | 1.05E-04 | 2.13 | 1.16E-06 | 2.92 | 9.41E-13 | 2.53 | 3.37E-13 |
| Il1rn | Interleukin 1 receptor antagonist (Il1rn), transcript variant 2 | | 2.5 | 1.18E-08 | 1.9 | 1.81E-04 | 2.11 | 1.43E-06 | 2.41 | 1.66E-08 | 2.23 | 8.41E-10 |
| Mlp | MARCKS-like 1 | | 2.46 | 3.68E-08 | 1.98 | 3.50E-05 | 2.18 | 3.06E-07 | 3.03 | 1.24E-13 | 2.31 | 2.69E-09 |
| Ltb | Lymphotoxin B | | 2.42 | 7.84E-08 | 2.42 | 6.46E-09 | 2.6 | 7.27E-11 | 2.95 | 4.90E-13 | 2.56 | 1.94E-13 |
| Errfi1 | ERBB receptor feedback inhibitor 1 | | 2.39 | 4.03E-09 | 1.96 | 3.50E-05 | 2.45 | 4.62E-12 | 2.8 | 8.72E-12 | 2.29 | 7.50E-11 |
| Ets2 | E26 avian leukemia oncogene 2, 3' domain | | 2.38 | 1.31E-12 | 2.14 | 7.36E-10 | 2.29 | 1.38E-11 | 2.5 | 3.75E-09 | 2.44 | 6.15E-15 |
| Ccl3 | Chemokine (C-C motif) ligand 3 | | 2.35 | 1.93E-13 | 2.14 | 2.64E-10 | 2.25 | 2.28E-11 | 2.45 | 6.67E-17 | 2.35 | 8.34E-14 |
| Zfp36 | Zinc finger protein 36 | | 2.26 | 1.04E-09 | 2.09 | 1.49E-08 | 2.89 | 3.36E-19 | 2.48 | 3.75E-09 | 2.27 | 1.14E-11 |
| Adora2b | Adenosine A2b receptor | | 2.2 | 5.39E-07 | 1.86 | 3.14E-04 | 2.13 | 1.24E-07 | 2.47 | 5.34E-09 | 2.08 | 3.05E-08 |
| Dusp2 | Dual specificity phosphatase 2 | | 2.11 | 3.43E-06 | 2.03 | 8.45E-06 | 2.77 | 4.06E-16 | 2.31 | 1.23E-07 | 2.26 | 1.96E-10 |
| Ccl9 | Chemokine (C-C motif) ligand 9 | | 2.08 | 4.82E-09 | 1.85 | 2.89E-06 | 2.01 | 7.33E-08 | 2.21 | 8.24E-10 | 2.14 | 6.13E-10 |
| Arhgef3 | Rho guanine nucleotide exchange factor (GEF) 3 | | 2.08 | 1.36E-05 | 1.59 | 3.15E-02 | 1.68 | 4.59E-03 | 2.32 | 9.56E-08 | 1.75 | 1.27E-04 |
| Csf2 | Colony stimulating factor 2 (granulocyte-macrophage) | | 2.05 | 4.85E-05 | 1.46 | >0.05 | 1.92 | 6.48E-05 | 2.64 | 2.18E-10 | 1.81 | 3.38E-04 |
| Rassf4 | Ras association (RalGDS/AF-6) domain family member 4 | | 2.02 | 6.55E-07 | 1.62 | 3.02E-02 | 1.68 | 2.45E-04 | 2.15 | 2.06E-06 | 1.87 | 1.56E-06 |

| Gene symbol | | Description | 1119-3 | | C10 | | C29 | | D6 | | D7 | |
| --- | --- | --- | --- | --- | --- | --- | --- | --- | --- | --- | --- | --- |
|  |  |  | FC | P-value | FC | P-value | FC | P-value | FC | P-value | FC | P-value |
| Signal transduction (*Continued*) | | |  |  |  |  |  |  |  |  |  |  |
| Grap | GRB2-related adaptor protein | | 2.0 | 1.18E-04 | 1.76 | 2.13E-03 | 1.79 | 8.04E-04 | 2.09 | 6.91E-06 | 1.87 | 1.05E-05 |
| Ccl4 | Chemokine (C-C motif) ligand 4 | | 1.97 | 9.80E-08 | 1.82 | 5.99E-06 | 1.98 | 1.67E-07 | 1.99 | 4.72E-07 | 1.99 | 4.53E-08 |
| Il1a | Interleukin 1 alpha | | 1.95 | 2.37E-04 | 1.43 | >0.05 | 1.66 | 6.70E-04 | 2.06 | 1.26E-05 | 1.71 | 2.49E-03 |
| Ikbke | Inhibitor of kappaB kinase epsilon | | 1.95 | 2.49E-04 | 1.71 | 4.53E-03 | 1.87 | 1.79E-04 | 2.25 | 3.52E-07 | 1.99 | 9.90E-07 |
| Rgs16 | Regulator of G-protein signaling 16 | | 1.94 | 2.73E-04 | 1.62 | 0.02 | 1.72 | 2.20E-03 | 1.98 | 5.35E-05 | 1.88 | 4.98E-05 |
| Relb | Avian reticuloendotheliosis viral (v-rel) oncogene related B | | 1.94 | 2.86E-04 | 1.87 | 2.68E-04 | 1.94 | 4.10E-05 | 2.09 | 7.15E-06 | 2.01 | 2.55E-07 |
| Plk2 | Polo-like kinase 2 (Drosophila) | | 1.91 | 7.08E-06 | 1.6 | 3.47E-03 | 1.92 | 3.75E-07 | 1.89 | 2.36E-04 | 1.74 | 4.43E-05 |
| Plk3 | Polo-like kinase 3 (Drosophila) | | 1.86 | 6.47E-04 | 1.66 | 0.01 | 1.75 | 1.05E-03 | 1.83 | 7.63E-04 | 1.81 | 2.86E-05 |
| Stap1 | Signal transducing adaptor family member 1 | | 1.86 | 1.25E-05 | 1.77 | 4.36E-05 | 1.83 | 5.82E-06 | 2.07 | 1.10E-05 | 1.93 | 1.22E-07 |
| Gadd45b | Growth arrest and DNA-damage-inducible 45 beta | | 1.8 | 2.45E-03 | 1.66 | 0.01 | 1.84 | 2.93E-04 | 1.99 | 4.77E-05 | 1.81 | 9.06E-05 |
| Rhoc | Ras homolog gene family, member C | | 1.8 | 7.67E-04 | 1.67 | 6.93E-03 | 1.78 | 2.00E-04 | 1.99 | 4.05E-05 | 1.92 | 1.39E-06 |
| Hbegf | Heparin-binding EGF-like growth factor | | 1.79 | 2.93E-03 | 1.56 | >0.05 | 1.66 | 6.08E-03 | 1.74 | 3.28E-03 | 1.77 | 1.79E-04 |
| Lilrb4 | Leukocyte immunoglobulin-like receptor, subfamily B, member 4 | | 1.77 | 3.46E-04 | 1.76 | 2.39E-04 | 1.92 | 5.40E-07 | 2.25 | 3.39E-07 | 2.11 | 3.16E-09 |
| Dusp16 | Dual specificity phosphatase 16, transcript variant A1 | | 1.75 | 6.04E-03 | 1.56 | >0.05 | 1.85 | 2.48E-04 | 1.88 | 2.74E-04 | 1.72 | 2.88E-04 |
| Tgfb1 | Transforming growth factor, beta 1 | | 1.73 | 9.58E-04 | 1.69 | 1.26E-03 | 1.67 | 4.50E-04 | 1.87 | 3.75E-04 | 1.85 | 4.37E-06 |
| Tlr2 | Toll-like receptor 2 | | 1.67 | 2.46E-04 | 1.77 | 1.48E-05 | 1.87 | 1.67E-06 | 2.05 | 1.73E-05 | 1.88 | 4.29E-07 |
| Irak2 | Interleukin-1 receptor-associated kinase 2 | | 1.66 | 2.30E-03 | 1.49 | 0.03 | 1.56 | 3.41E-03 | 1.7 | 6.13-E03 | 1.65 | 5.53E-04 |

| Gene symbol | | Description | 1119-3 | | C10 | | C29 | | D6 | | D7 | |
| --- | --- | --- | --- | --- | --- | --- | --- | --- | --- | --- | --- | --- |
|  |  |  | FC | P-value | FC | P-value | FC | P-value | FC | P-value | FC | P-value |
| Signal transduction (*Continued*) | | |  |  |  |  |  |  |  |  |  |  |
| Ebi3 | Epstein-Barr virus induced gene 3 | | 1.65 | 0.01 | 1.41 | >0.05 | 1.49 | >0.05 | 1.68 | 8.04E-03 | 1.51 | 0.02 |
| Dtx4 | Deltex 4 homolog (Drosophila) | | 1.65 | 0.01 | 1.62 | 0.01 | 1.65 | 3.20E-03 | 1.86 | 3.93E-04 | 1.82 | 2.05E-05 |
| Pip5k1a | Phosphatidylinositol-4-phosphate 5-kinase, type 1 alpha | | 1.63 | 4.17E-03 | 1.39 | >0.05 | 1.52 | 0.01 | 1.35 | >0.05 | 1.28 | >0.05 |
| Dusp4 | Dual specificity phosphatase 4 | | 1.61 | 4.93E-03 | 1.52 | 0.02 | 1.71 | 9.45E-05 | 1.63 | 0.02 | 1.6 | 1.30E-03 |
| Rgl1 | Ral guanine nucleotide dissociation stimulator,-like 1 | | 1.6 | 0.04 | 1.47 | >0.05 | 1.49 | >0.05 | 1.55 | >0.05 | 1.58 | 5.15E-03 |
| Mapkapk2 | MAP kinase-activated protein kinase 2 | | 1.56 | 0.01 | 1.5 | 0.03 | 1.48 | 0.02 | 1.69 | 7.55E-03 | 1.7 | 1.27E-04 |
| Ptpn12 | Protein tyrosine phosphatase, non-receptor type 12 | | 1.52 | >0.05 | 1.39 | >0.05 | 1.54 | 0.04 | 1.51 | >0.05 | 1.46 | >0.05 |
| Btg1 | B-cell translocation gene 1, anti-proliferative | | 1.51 | 0.03 | 1.41 | >0.05 | 1.54 | 5.02E-03 | 1.56 | 0.04 | 1.46 | 0.03 |
| Rel | Reticuloendotheliosis oncogene | | 1.49 | >0.05 | 1.38 | >0.05 | 1.72 | 2.60E-03 | 1.66 | 0.01 | 1.51 | 0.02 |
| Cd82 | CD82 antigen (Cd82), transcript variant 1 | | 1.41 | >0.05 | 1.58 | 0.01 | 1.57 | 4.14E-03 | 1.65 | 0.01 | 1.56 | 4.91E-03 |
| Adrb2 | Adrenergic receptor, beta 2 | | 1.37 | >0.05 | 1.4 | >0.05 | 1.53 | 0.04 | 1.44 | >0.05 | 1.42 | >0.05 |
| Immunity and defense | | |  |  |  |  |  |  |  |  |  |  |
| Cxcl2 | Chemokine (C-X-C motif) ligand 2 | | 34.68 | 1.56E-193 | 21.51 | 1.54E-156 | 33.41 | 7.14E-220 | 43.82 | 5.67E-166 | 29.62 | 4.21E-205 |
| Tnf | Tumor necrosis factor | | 17.47 | 4.45E-150 | 14.18 | 3.82E-133 | 21.83 | 1.58E-168 | 22.45 | 6.39E-120 | 18.35 | 3.46E-157 |
| Ier3 | Immediate early response 3 | | 8.35 | 4.93E-82 | 6.36 | 1.01E-63 | 9.03 | 7.44E-87 | 9.04 | 1.02E-55 | 7.6 | 2.81E-78 |
| Ccl2 | Chemokine (C-C motif) ligand 2 | | 6.16 | 4.74E-50 | 4.03 | 1.50E-31 | 5.2 | 2.46E-48 | 6.16 | 9.30E-38 | 5.14 | 3.57E-47 |
| Ccl7 | Chemokine (C-C motif) ligand 7 | | 5.26 | 8.97E-32 | 3.48 | 2.07E-18 | 3.68 | 7.03E-21 | 5.91 | 5.23E-36 | 4.85 | 1.48E-39 |
| Il1b | Interleukin 1 beta | | 4.89 | 2.78E-26 | 2.87 | 5.56E-13 | 3.63 | 2.88E-20 | 6.12 | 1.95E-37 | 4.29 | 6.18E-33 |
| Gene symbol | | Description | 1119-3 | | C10 | | C29 | | D6 | | D7 | |
|  |  |  | FC | P-value | FC | P-value | FC | P-value | FC | P-value | FC | P-value |
| Immunity and defense (*Continued*) | | |  |  |  |  |  |  |  |  |  |  |
| Cish | Cytokine inducible SH2-containing protein | | 4.43 | 4.39E-23 | 3.0 | 3.85E-14 | 3.83 | 6.24E-22 | 5.35 | 5.20E-32 | 4.21 | 2.63E-32 |
| Nfkbia | Nuclear factor of kappa light polypeptide gene enhancer in B-cells inhibitor, alpha | | 3.58 | 1.78E-22 | 3.34 | 2.00E-21 | 4.25 | 1.50E-36 | 4.46 | 2.12E-25 | 3.73 | 2.96E-29 |
| Clecsf9 | C-type lectin domain family 4, member e | | 3.11 | 2.96E-20 | 2.9 | 5.21E-19 | 2.82 | 2.50E-18 | 3.57 | 4.49E-18 | 3.23 | 2.54E-25 |
| Ccrl2 | Chemokine (C-C motif) receptor-like 2 | | 2.93 | 1.09E-11 | 2.41 | 6.64E-09 | 2.87 | 2.58E-13 | 3.61 | 1.73E-18 | 2.74 | 2.19E-15 |
| Cd40 | CD40 antigen, transcript variant 5 | | 2.73 | 3.72E-10 | 2.13 | 1.99E-06 | 2.23 | 1.37E-07 | 2.97 | 3.40E-13 | 2.45 | 1.60E-11 |
| Icam1 | Intercellular adhesion molecule 1 | | 2.54 | 1.15E-12 | 2.52 | 1.27E-13 | 2.76 | 1.25E-17 | 3.0 | 1.94E-13 | 2.76 | 5.20E-18 |
| Il13ra2 | Interleukin 13 receptor, alpha 2 | | 2.53 | 1.08E-08 | 2.01 | 1.91E-05 | 2.29 | 3.80E-08 | 2.81 | 8.06E-12 | 2.49 | 1.74E-11 |
| Tnfrsf1b | Tumor necrosis factor receptor superfamily, member 1b | | 2.51 | 1.57E-08 | 1.92 | 1.05E-04 | 2.13 | 1.16E-06 | 2.92 | 9.41E-13 | 2.53 | 3.37E-13 |
| Il1rn | Interleukin 1 receptor antagonist, transcript variant 2 | | 2.5 | 1.18E-08 | 1.9 | 1.81E-04 | 2.11 | 1.43E-06 | 2.41 | 1.66E-08 | 2.23 | 8.41E-10 |
| Ltb | Lymphotoxin B | | 2.42 | 7.84E-08 | 2.42 | 6.46E-09 | 2.6 | 7.27E-11 | 2.95 | 4.90E-13 | 2.56 | 1.94E-13 |
| Plaur | Plasminogen activator, urokinase receptor | | 2.42 | 3.98E-11 | 2.0 | 3.69E-07 | 2.1 | 2.90E-09 | 2.4 | 2.27E-08 | 2.22 | 1.23E-10 |
| Clec4e | C-type lectin domain family 4, member e | | 2.41 | 4.47E-11 | 2.0 | 3.07E-07 | 2.17 | 2.65E-10 | 2.57 | 7.81E-10 | 2.42 | 3.25E-13 |
| Ccl3 | Chemokine (C-C motif) ligand 3 | | 2.35 | 1.93E-13 | 2.14 | 2.64E-10 | 2.25 | 2.28E-11 | 2.45 | 6.67E-17 | 2.35 | 8.34E-14 |
| Tnfaip2 | Tumor necrosis factor, alpha-induced protein 2 | | 2.34 | 2.73E-10 | 1.98 | 5.43E-07 | 2.23 | 5.86E-11 | 2.53 | 1.76E-09 | 2.27 | 2.73E-11 |
| Lcn2 | Lipocalin 2 | | 2.21 | 3.36E-06 | 1.36 | >0.05 | 1.66 | 5.97E-03 | 2.66 | 1.49E-10 | 1.83 | 2.71E-0.4 |
| Adora2b | Adenosine A2b receptor | | 2.2 | 5.39E-07 | 1.86 | 3.14E-04 | 2.13 | 1.24E-07 | 2.47 | 5.34E-09 | 2.08 | 3.05E-08 |
| Ccl9 | Chemokine (C-C motif) ligand 9 | | 2.08 | 4.82E-09 | 1.85 | 2.89E-06 | 2.01 | 7.33E-08 | 2.21 | 8.24E-10 | 2.14 | 6.13E-10 |

| Gene symbol | | Description | 1119-3 | | C10 | | C29 | | D6 | | D7 | |
| --- | --- | --- | --- | --- | --- | --- | --- | --- | --- | --- | --- | --- |
|  |  |  | FC | P-value | FC | P-value | FC | P-value | FC | P-value | FC | P-value |
| Immunity and defense (*Continued*) | | |  |  |  |  |  |  |  |  |  |  |
| Gadd45a | Growth arrest and DNA-damage-inducible 45 alpha | | 2.06 | 4.13E-05 | 1.86 | 3.25E-04 | 2.19 | 3.43E-07 | 2.47 | 5.81E-09 | 2.12 | 2.93E-08 |
| Csf2 | Colony stimulating factor 2 (granulocyte-macrophage) | | 2.05 | 4.85E-05 | 1.46 | >0.05 | 1.92 | 6.48E-05 | 2.64 | 2.18E-10 | 1.81 | 3.38E-04 |
| Saa3 | Serum amyloid A 3 | | 2.01 | 9.56E-05 | 1.53 | >0.05 | 1.64 | 9.39E-03 | 2.34 | 5.98E-08 | 1.67 | 1.95E-03 |
| Grap | GRB2-related adaptor protein | | 2.0 | 1.18E-04 | 1.76 | 2.13E-03 | 1.79 | 8.04E-04 | 2.09 | 6.91E-06 | 1.87 | 1.05E-05 |
| Ccl4 | Chemokine (C-C motif) ligand 4 | | 1.97 | 9.80E-08 | 1.82 | 5.99E-06 | 1.98 | 1.67E-07 | 1.99 | 4.72E-07 | 1.99 | 4.53E-08 |
| Il1a | Interleukin 1 alpha | | 1.95 | 2.37E-04 | 1.43 | >0.05 | 1.66 | 6.70E-04 | 2.06 | 1.26E-05 | 1.71 | 2.49E-03 |
| Ikbke | Inhibitor of kappaB kinase epsilon | | 1.95 | 2.49E-04 | 1.71 | 4.53E-03 | 1.87 | 1.79E-04 | 2.25 | 3.52E-07 | 1.99 | 9.90E-07 |
| Relb | Avian reticuloendotheliosis viral (v-rel) oncogene related B | | 1.94 | 2.86E-04 | 1.87 | 2.68E-04 | 1.94 | 4.10E-05 | 2.09 | 7.15E-06 | 2.01 | 2.55E-07 |
| Sqstm1 | Sequestosome 1 | | 1.81 | 7.85E-06 | 1.72 | 6.01E-05 | 1.83 | 4.39E-06 | 1.94 | 1.11E-04 | 1.9 | 2.55E-07 |
| Gadd45b | Growth arrest and DNA-damage-inducible 45 beta | | 1.8 | 2.45E-03 | 1.66 | 0.01 | 1.84 | 2.93E-04 | 1.99 | 4.77E-05 | 1.81 | 9.06E-05 |
| C3 | Complement component 3 | | 1.78 | 3.60E-03 | 1.54 | >0.05 | 1.64 | 8.57E-03 | 1.86 | 3.83E-04 | 1.57 | 0.02 |
| Dusp16 | Dual specificity phosphatase 16, transcript variant A1 | | 1.75 | 6.04E-03 | 1.56 | >0.05 | 1.85 | 2.48E-04 | 1.88 | 2.74E-04 | 1.72 | 2.88E-04 |
| Cd14 | CD14 antigen | | 1.68 | 2.36E-04 | 1.51 | 0.01 | 1.63 | 8.87E-04 | 1.76 | 1.21E-03 | 1.69 | 1.71E-04 |
| Sdc4 | Syndecan 4 | | 1.66 | 0.02 | 1.46 | >0.05 | 1.69 | 3.15E-03 | 1.82 | 7.79E-04 | 1.74 | 1.38E-04 |
| Irak2 | Interleukin-1 receptor-associated kinase 2 | | 1.66 | 2.29E-03 | 1.49 | 0.03 | 1.56 | 3.41E-03 | 1.7 | 6.13E-03 | 1.65 | 5.53E-04 |
| Ebi3 | Epstein-Barr virus induced gene 3 | | 1.65 | 0.01 | 1.41 | >0.05 | 1.49 | >0.05 | 1.68 | 8.04E-03 | 1.51 | 0.02 |
| Mapkapk2 | MAP kinase-activated protein kinase 2 | | 1.56 | 0.01 | 1.5 | 0.03 | 1.48 | 0.02 | 1.69 | 7.55E-03 | 1.7 | 1.27E-04 |

| Gene symbol | | Description | 1119-3 | | C10 | | C29 | | D6 | | D7 | |
| --- | --- | --- | --- | --- | --- | --- | --- | --- | --- | --- | --- | --- |
|  |  |  | FC | P-value | FC | P-value | FC | P-value | FC | P-value | FC | P-value |
| Immunity and defense (*Continued*) | | |  |  |  |  |  |  |  |  |  |  |
| Rel | Reticuloendotheliosis oncogene | | 1.49 | >0.05 | 1.38 | >0.05 | 1.72 | 2.60E-03 | 1.66 | 0.01 | 1.51 | 0.02 |
| Cd82 | CD82 antigen (Cd82), transcript variant 1 | | 1.41 | >0.05 | 1.58 | 0.01 | 1.57 | 4.14E-03 | 1.65 | 0.01 | 1.56 | 4.91E-03 |
| Apoptosis | | |  |  |  |  |  |  |  |  |  |  |
| Tnf | Tumor necrosis factor | | 17.47 | 4.45E-150 | 14.18 | 3.82E-133 | 21.83 | 1.58E-168 | 22.45 | 6.39E-120 | 18.35 | 3.46E-157 |
| Il1b | Interleukin 1 beta | | 4.89 | 2.78E-26 | 2.87 | 5.56E-13 | 3.63 | 2.88E-20 | 6.12 | 1.95E-37 | 4.29 | 6.18E-33 |
| Cish | Cytokine inducible SH2-containing protein | | 4.43 | 4.39E-23 | 3.0 | 3.85E-14 | 3.83 | 6.24E-22 | 5.35 | 5.20E-32 | 4.21 | 2.63E-32 |
| Socs3 | Suppressor of cytokine signaling 3 | | 3.66 | 1.95E-20 | 2.45 | 3.55E-09 | 3.78 | 1.80E-26 | 4.93 | 6.06E-29 | 3.05 | 1.49E-19 |
| Nfkbia | Nuclear factor of kappa light polypeptide gene enhancer in B-cells inhibitor, alpha | | 3.58 | 1.78E-22 | 3.34 | 2.00E-21 | 4.25 | 1.50E-36 | 4.46 | 2.12E-25 | 3.73 | 2.96E-29 |
| Fas | Fas (TNF receptor superfamily member 6) | | 2.51 | 1.55E-08 | 2.11 | 3.06E-06 | 2.21 | 2.21E-07 | 2.82 | 7.05E-12 | 2.47 | 5.30E-12 |
| Adora2b | Adenosine A2b receptor | | 2.2 | 5.39E-07 | 1.86 | 3.14E-04 | 2.13 | 1.24E-07 | 2.47 | 5.34E-09 | 2.08 | 3.05E-08 |
| Atf3 | Activating transcription factor 3 | | 2.19 | 1.00E-08 | 1.88 | 5.14E-06 | 2.21 | 8.81E-11 | 2.25 | 3.52E-07 | 2.1 | 3.03E-09 |
| Arhgef3 | Rho guanine nucleotide exchange factor (GEF) 3 | | 2.08 | 1.36E-05 | 1.59 | 3.15E-02 | 1.68 | 4.59E-03 | 2.32 | 9.56E-08 | 1.75 | 1.27E-04 |
| Gadd45a | Growth arrest and DNA-damage-inducible 45 alpha | | 2.06 | 4.13E-05 | 1.86 | 3.25E-04 | 2.19 | 3.43E-07 | 2.47 | 5.81E-09 | 2.12 | 2.93E-08 |
| Rassf4 | Ras association (RalGDS/AF-6) domain family member 4 | | 2.02 | 6.55E-07 | 1.62 | 3.02E-02 | 1.68 | 2.45E-04 | 2.15 | 2.06E-06 | 1.87 | 1.56E-06 |
| Nlrp3 | NLR family, pyrin domain containing 3 | | 1.99 | 1.37E-04 | 1.78 | 1.35E-03 | 2.07 | 2.96E-06 | 2.34 | 6.55E-08 | 2.07 | 6.60E-07 |
| Relb | Avian reticuloendotheliosis viral (v-rel) oncogene related B | | 1.94 | 2.86E-04 | 1.87 | 2.68E-04 | 1.94 | 4.10E-05 | 2.09 | 7.15E-06 | 2.01 | 2.55E-07 |
| Cias1 | Cold autoinflammatory syndrome 1 homolog (human) | | 1.86 | 1.03E-03 | 1.61 | 0.02 | 1.83 | 3.62E-04 | 1.91 | 1.72E-04 | 1.76 | 7.20E-04 |

| Gene symbol | | Description | 1119-3 | | C10 | | C29 | | D6 | | D7 | |
| --- | --- | --- | --- | --- | --- | --- | --- | --- | --- | --- | --- | --- |
|  |  |  | FC | P-value | FC | P-value | FC | P-value | FC | P-value | FC | P-value |
| Apoptosis (*Continued*) | | |  |  |  |  |  |  |  |  |  |  |
| Gadd45b | Growth arrest and DNA-damage-inducible 45 beta | | 1.8 | 2.45E-03 | 1.66 | 0.01 | 1.84 | 2.93E-04 | 1.99 | 4.77E-05 | 1.81 | 9.06E-05 |
| Bcl2a1d | B cell leukemia/lymphoma 2 related protein A1d | | 1.78 | 1.12E-04 | 1.6 | 3.13E-03 | 1.64 | 5.56E-04 | 1.94 | 1.02E-04 | 1.75 | 2.49E-05 |
| Casp4 | Caspase 4, apoptosis-related cysteine peptidase | | 1.74 | 6.85E-03 | 1.52 | >0.05 | 1.65 | 8.57E-03 | 1.69 | 7.11E-03 | 1.59 | 5.44E-03 |
| Bcl2a1b | B-cell leukemia/lymphoma 2 related protein A1b | | 1.62 | 2.45E-03 | 1.51 | 0.02 | 1.41 | >0.05 | 1.56 | 0.04 | 1.37 | >0.05 |
| Rel | Reticuloendotheliosis oncogene | | 1.49 | >0.05 | 1.38 | >0.05 | 1.72 | 2.60E-03 | 1.66 | 0.01 | 1.51 | 0.02 |
| Cell proliferation and differentiation | | |  |  |  |  |  |  |  |  |  |  |
| Cxcl2 | Chemokine (C-X-C motif) ligand 2 | | 34.68 | 1.56E-193 | 21.51 | 1.54E-156 | 33.41 | 7.14E-220 | 43.82 | 5.67E-166 | 29.62 | 4.21E-205 |
| Il1b | Interleukin 1 beta | | 4.89 | 2.78E-26 | 2.87 | 5.56E-13 | 3.63 | 2.88E-20 | 6.12 | 1.95E-37 | 4.29 | 6.18E-33 |
| Nfkbia | Nuclear factor of kappa light polypeptide gene enhancer in B-cells inhibitor, alpha | | 3.58 | 1.78E-22 | 3.34 | 2.00E-21 | 4.25 | 1.50E-36 | 4.46 | 2.12E-25 | 3.73 | 2.96E-29 |
| Edn1 | Endothelin 1 | | 3.53 | 2.85E-16 | 2.28 | 8.95E-08 | 2.49 | 5.74E-10 | 3.51 | 1.20E-17 | 2.84 | 1.34E-15 |
| Vegfc | Vascular endothelial growth factor C | | 2.77 | 1.05E-10 | 2.36 | 2.38E-08 | 2.27 | 5.94E-08 | 3.03 | 1.18E-13 | 2.63 | 2.16E-14 |
| Il13ra2 | Interleukin 13 receptor, alpha 2 | | 2.53 | 1.08E-08 | 2.01 | 1.91E-05 | 2.29 | 3.80E-08 | 2.81 | 8.06E-12 | 2.49 | 1.74E-11 |
| Errfi1 | ERBB receptor feedback inhibitor 1 | | 2.39 | 4.03E-09 | 1.96 | 3.50E-05 | 2.45 | 4.62E-12 | 2.8 | 8.72E-12 | 2.29 | 7.50E-11 |
| Ets2 | E26 avian leukemia oncogene 2, 3' domain | | 2.38 | 1.31E-12 | 2.14 | 7.36E-10 | 2.29 | 1.38E-11 | 2.5 | 3.75E-09 | 2.44 | 6.15E-15 |
| Il1a | Interleukin 1 alpha | | 1.95 | 2.37E-04 | 1.43 | >0.05 | 1.66 | 6.70E-04 | 2.06 | 1.26E-05 | 1.71 | 2.49E-03 |
| Relb | Avian reticuloendotheliosis viral (v-rel) oncogene related B | | 1.94 | 2.86E-04 | 1.87 | 2.68E-04 | 1.94 | 4.10E-05 | 2.09 | 7.15E-06 | 2.01 | 2.55E-07 |
| Myd116 | Myeloid differentiation primary response gene 116 | | 1.93 | 1.14E-04 | 1.56 | >0.05 | 1.91 | 1.98E-05 | 2.0 | 3.88E-05 | 1.75 | 1.16E-04 |

| Gene symbol | | Description | 1119-3 | | C10 | | C29 | | D6 | | D7 | |
| --- | --- | --- | --- | --- | --- | --- | --- | --- | --- | --- | --- | --- |
|  |  |  | FC | P-value | FC | P-value | FC | P-value | FC | P-value | FC | P-value |
| Cell proliferation and differentiation (*Continued*) | | |  |  |  |  |  |  |  |  |  |  |
| Plk2 | Polo-like kinase 2 (Drosophila) | | 1.91 | 7.08E-06 | 1.6 | 3.47E-03 | 1.92 | 3.75E-07 | 1.89 | 2.36E-04 | 1.74 | 4.43E-05 |
| Plk3 | Polo-like kinase 3 (Drosophila) | | 1.86 | 6.47E-04 | 1.66 | 0.01 | 1.75 | 1.05E-03 | 1.83 | 7.63E-04 | 1.81 | 2.86E-05 |
| Stap1 | Signal transducing adaptor family member 1 | | 1.86 | 1.25E-05 | 1.77 | 4.36E-05 | 1.83 | 5.82E-06 | 2.07 | 1.10E-05 | 1.93 | 1.22E-07 |
| Gadd45b | Growth arrest and DNA-damage-inducible 45 beta | | 1.8 | 2.45E-03 | 1.66 | 0.01 | 1.84 | 2.93E-04 | 1.99 | 4.77E-05 | 1.81 | 9.06E-05 |
| Hbegf | Heparin-binding EGF-like growth factor | | 1.79 | 2.93E-03 | 1.56 | >0.05 | 1.66 | 6.08E-03 | 1.74 | 3.28E-03 | 1.77 | 1.79E-04 |
| Tgfb1 | Transforming growth factor, beta 1 | | 1.73 | 9.58E-04 | 1.69 | 1.26E-03 | 1.67 | 4.50E-04 | 1.87 | 3.75E-04 | 1.85 | 4.37E-06 |
| Cdkn1a | Cyclin-dependent kinase inhibitor 1A (P21) | | 1.62 | 6.04E-03 | 1.53 | 0.02 | 1.6 | 1.97E-03 | 1.7 | 6.13E-03 | 1.6 | 7.51E-04 |
| Btg1 | B-cell translocation gene 1, anti-proliferative | | 1.51 | 0.03 | 1.41 | >0.05 | 1.54 | 5.02E-03 | 1.56 | 0.04 | 1.46 | 0.03 |
| Rel | Reticuloendotheliosis oncogene | | 1.49 | >0.05 | 1.38 | >0.05 | 1.72 | 2.60E-03 | 1.66 | 0.01 | 1.51 | 0.02 |
| Developmental processes | | |  |  |  |  |  |  |  |  |  |  |
| Cish | Cytokine inducible SH2-containing protein | | 4.43 | 4.39E-23 | 3.0 | 3.85E-14 | 3.83 | 6.24E-22 | 5.35 | 5.20E-32 | 4.21 | 2.63E-32 |
| Socs3 | Suppressor of cytokine signaling 3 | | 3.66 | 1.95E-20 | 2.45 | 3.55E-09 | 3.78 | 1.80E-26 | 4.93 | 6.06E-29 | 3.05 | 1.49E-19 |
| Vegfc | Vascular endothelial growth factor C | | 2.77 | 1.05E-10 | 2.36 | 2.38E-08 | 2.27 | 5.94E-08 | 3.03 | 1.18E-13 | 2.63 | 2.16E-14 |
| Tnfrsf1b | Tumor necrosis factor receptor superfamily, member 1b | | 2.51 | 1.57E-08 | 1.92 | 1.05E-04 | 2.13 | 1.16E-06 | 2.92 | 9.41E-13 | 2.53 | 3.37E-13 |
| Ets2 | E26 avian leukemia oncogene 2, 3' domain | | 2.38 | 1.31E-12 | 2.14 | 7.36E-10 | 2.29 | 1.38E-11 | 2.5 | 3.75E-09 | 2.44 | 6.15E-15 |
| Tnfaip2 | Tumor necrosis factor, alpha-induced protein 2 | | 2.34 | 2.73E-10 | 1.98 | 5.43E-07 | 2.23 | 5.86E-11 | 2.53 | 1.76E-09 | 2.27 | 2.73E-11 |
| Adora2b | Adenosine A2b receptor | | 2.2 | 5.39E-07 | 1.86 | 3.14E-04 | 2.13 | 1.24E-07 | 2.47 | 5.34E-09 | 2.08 | 3.05E-08 |
| Pou2f2 | POU domain, class 2, transcription factor 2 | | 2.15 | 8.50E-06 | 1.94 | 7.04E-05 | 2.05 | 4.91E-06 | 2.38 | 3.15E-08 | 2.1 | 6.71E-08 |

| Gene symbol | | Description | 1119-3 | | C10 | | C29 | | D6 | | D7 | |
| --- | --- | --- | --- | --- | --- | --- | --- | --- | --- | --- | --- | --- |
|  |  |  | FC | P-value | FC | P-value | FC | P-value | FC | P-value | FC | P-value |
| Developmental processes (*Continued*) | | |  |  |  |  |  |  |  |  |  |  |
| Dusp2 | Dual specificity phosphatase 2 | | 2.11 | 3.43E-06 | 2.03 | 8.45E-06 | 2.77 | 4.06E-16 | 2.31 | 1.23E-07 | 2.26 | 1.96E-10 |
| Plagl2 | Pleiomorphic adenoma gene-like 2 | | 1.93 | 4.95E-06 | 1.6 | 3.76E-03 | 1.8 | 9.01E-06 | 2.06 | 1.25E-05 | 1.84 | 3.21E-06 |
| Plk2 | Polo-like kinase 2 (Drosophila) | | 1.91 | 7.08E-06 | 1.6 | 3.47E-03 | 1.92 | 3.75E-07 | 1.89 | 2.36E-04 | 1.74 | 4.43E-05 |
| Plk3 | Polo-like kinase 3 (Drosophila) | | 1.86 | 6.47E-04 | 1.66 | 0.01 | 1.75 | 1.05E-03 | 1.83 | 7.63E-04 | 1.81 | 2.86E-05 |
| Slfn2 | Schlafen 2 | | 1.84 | 1,57E-03 | 1.86 | 3.26E-04 | 2.15 | 6.38E-07 | 2.16 | 1.82E-06 | 2.13 | 4.49E-08 |
| Tgfb1 | Transforming growth factor, beta 1 | | 1.73 | 9.58E-04 | 1.69 | 1.26E-03 | 1.67 | 4.50E-04 | 1.87 | 3.75E-04 | 1.85 | 4.37E-06 |
| Tlr2 | Toll-like receptor 2 | | 1.67 | 2.46E-04 | 1.77 | 1.48E-05 | 1.87 | 1.67E-06 | 2.05 | 1.73E-05 | 1.88 | 4.29E-07 |
| Sdc4 | Syndecan 4 | | 1.66 | 0.02 | 1.46 | >0.05 | 1.69 | 3.15E-03 | 1.82 | 7.79E-04 | 1.74 | 1.38E-04 |
| Dtx4 | Deltex 4 homolog (Drosophila) | | 1.65 | 0.01 | 1.62 | 0.01 | 1.65 | 3.20E-03 | 1.86 | 3.93E-04 | 1.82 | 2.05E-05 |
| Dusp4 | Dual specificity phosphatase 4 | | 1.61 | 4.93E-03 | 1.52 | 0.02 | 1.71 | 9.45E-05 | 1.63 | 0.02 | 1.6 | 1.30E-03 |
| BC003324 | Rab interacting lysosomal protein-like 2 | | 1.59 | 0.04 | 1.49 | >0.05 | 1.36 | >0.05 | 1.39 | >0.05 | 1.39 | >0.05 |
| Rilpl2 | Rab interacting lysosomal protein-like 2 | | 1.53 | 0.02 | 1.46 | >0.05 | 1.37 | >0.05 | 1.41 | >0.05 | 1.4 | >0.05 |
| Rel | Reticuloendotheliosis oncogene | | 1.49 | >0.05 | 1.38 | >0.05 | 1.72 | 2.60E-03 | 1.66 | 0.01 | 1.51 | 0.02 |
| Cited2 | Cbp/p300-interacting transactivator, with Glu/Asp-rich carboxy-terminal domain, 2 | | 1.39 | >0.05 | 1.34 | >0.05 | 1.48 | 0.02 | 1.56 | 0.04 | 1.48 | 0.02 |
| Egr2 | Early growth response 2 | | 1.31 | >0.05 | 1.34 | >0.05 | 1.67 | 5.92E-03 | 1.44 | >0.05 | 1.45 | >0.05 |
| Cell cycle | | |  |  |  |  |  |  |  |  |  |  |
| Il1b | Interleukin 1 beta | | 4.89 | 2.78E-26 | 2.87 | 5.56E-13 | 3.63 | 2.88E-20 | 6.12 | 1.95E-37 | 4.29 | 6.18E-33 |

| Gene symbol | | Description | 1119-3 | | C10 | | C29 | | D6 | | D7 | |
| --- | --- | --- | --- | --- | --- | --- | --- | --- | --- | --- | --- | --- |
|  |  |  | FC | P-value | FC | P-value | FC | P-value | FC | P-value | FC | P-value |
| Cell cycle (*Continued*) | | |  |  |  |  |  |  |  |  |  |  |
| Vegfc | Vascular endothelial growth factor C | | 2.77 | 1.05E-10 | 2.36 | 2.38E-08 | 2.27 | 5.94E-08 | 3.03 | 1.18E-13 | 2.63 | 2.16E-14 |
| Dusp2 | Dual specificity phosphatase 2 | | 2.11 | 3.43E-06 | 2.03 | 8.45E-06 | 2.77 | 4.06E-16 | 2.31 | 1.23E-07 | 2.26 | 1.96E-10 |
| Arhgef3 | Rho guanine nucleotide exchange factor (GEF) 3 | | 2.08 | 1.36E-05 | 1.59 | 3.15E-02 | 1.68 | 4.59E-03 | 2.32 | 9.56E-08 | 1.75 | 1.27E-04 |
| Gadd45a | Growth arrest and DNA-damage-inducible 45 alpha | | 2.06 | 4.13E-05 | 1.86 | 3.25E-04 | 2.19 | 3.43E-07 | 2.47 | 5.81E-09 | 2.12 | 2.93E-08 |
| Il1a | Interleukin 1 alpha | | 1.95 | 2.37E-04 | 1.43 | >0.05 | 1.66 | 6.70E-04 | 2.06 | 1.26E-05 | 1.71 | 2.49E-03 |
| Slfn2 | Schlafen 2 | | 1.84 | 1,57E-03 | 1.86 | 3.26E-04 | 2.15 | 6.38E-07 | 2.16 | 1.82E-06 | 2.13 | 4.49E-08 |
| Gadd45b | Growth arrest and DNA-damage-inducible 45 beta | | 1.8 | 2.45E-03 | 1.66 | 0.01 | 1.84 | 2.93E-04 | 1.99 | 4.77E-05 | 1.81 | 9.06E-05 |
| Cdkn1a | Cyclin-dependent kinase inhibitor 1A (P21) | | 1.62 | 6.04E-03 | 1.53 | 0.02 | 1.6 | 1.97E-03 | 1.7 | 6.13E-03 | 1.6 | 7.51E-04 |
| Dusp4 | Dual specificity phosphatase 4 | | 1.61 | 4.93E-03 | 1.52 | 0.02 | 1.71 | 9.45E-05 | 1.63 | 0.02 | 1.6 | 1.30E-03 |
| Zyx | Zyxin | | 1.57 | 6.85E-03 | 1.42 | >0.05 | 1.46 | 0.03 | 1.45 | >0.05 | 1.54 | 3.41E-03 |
| Btg1 | B-cell translocation gene 1, anti-proliferative | | 1.51 | 0.03 | 1.41 | >0.05 | 1.54 | 5.02E-03 | 1.56 | 0.04 | 1.46 | 0.03 |
| Rel | Reticuloendotheliosis oncogene | | 1.49 | >0.05 | 1.38 | >0.05 | 1.72 | 2.60E-03 | 1.66 | 0.01 | 1.51 | 0.02 |
| Mak16 | MAK16 homolog (S. cerevisiae) | | 1.41 | >0.05 | 1.33 | >0.05 | 1.49 | 0.02 | 1.57 | 0.04 | 1.46 | 0.03 |
| Cell structure and motility | | |  |  |  |  |  |  |  |  |  |  |
| Cxcl2 | Chemokine (C-X-C motif) ligand 2 | | 34.68 | 1.56E-193 | 21.51 | 1.54E-156 | 33.41 | 7.14E-220 | 43.82 | 5.67E-166 | 29.62 | 4.21E-205 |
| Ccrl2 | Chemokine (C-C motif) receptor-like 2 | | 2.93 | 1.09E-11 | 2.41 | 6.64E-09 | 2.87 | 2.58E-13 | 3.61 | 1.73E-18 | 2.74 | 2.19E-15 |
| Arhgef3 | Rho guanine nucleotide exchange factor (GEF) 3 | | 2.08 | 1.36E-05 | 1.59 | 3.15E-02 | 1.68 | 4.59E-03 | 2.32 | 9.56E-08 | 1.75 | 1.27E-04 |
| Cdc42ep2 | CDC42 effector protein (Rho GTPase binding) 2 | | 2.0 | 1.09E-04 | 1.8 | 1.00E-03 | 2.09 | 2.32E-06 | 2.29 | 1.70E-07 | 1.78 | 2.94E-04 |

| Gene symbol | | Description | 1119-3 | | C10 | | C29 | | D6 | | D7 | |
| --- | --- | --- | --- | --- | --- | --- | --- | --- | --- | --- | --- | --- |
|  |  |  | FC | P-value | FC | P-value | FC | P-value | FC | P-value | FC | P-value |
| Cell structure and motility (*Continued*) | | |  |  |  |  |  |  |  |  |  |  |
| Rhoc | Ras homolog gene family, member C | | 1.8 | 7.67E-04 | 1.67 | 6.93E-03 | 1.78 | 2.00E-04 | 1.99 | 4.05E-05 | 1.92 | 1.39E-06 |
| Cdc42ep4 | CDC42 effector protein (Rho GTPase binding) 4 | | 1.79 | 1.88E-03 | 1.57 | 0.04 | 1.9 | 3.75E-05 | 1.9 | 1.99E-04 | 1.73 | 1.75E-04 |
| Lima1 | LIM domain and actin binding 1 | | 1.66 | 0.02 | 1.49 | >0.05 | 1.64 | 8.21E-03 | 1.62 | 0.02 | 1.48 | 0.03 |
| Zyx | Zyxin | | 1.57 | 6.85E-03 | 1.42 | >0.05 | 1.46 | 0.03 | 1.45 | >0.05 | 1.54 | 3.41E-03 |
| Ptpn12 | Protein tyrosine phosphatase, non-receptor type 12 | | 1.52 | >0.05 | 1.39 | >0.05 | 1.54 | 0.04 | 1.51 | >0.05 | 1.46 | >0.05 |
| Cdc42ep3 | CDC42 effector protein (Rho GTPase binding) 3 | | 1.38 | >0.05 | 1.27 | >0.05 | 1.47 | >0.05 | 1.46 | >0.05 | 1.5 | 0.02 |
| Oncogenesis | | |  |  |  |  |  |  |  |  |  |  |
| Ets2 | E26 avian leukemia oncogene 2, 3' domain | | 2.38 | 1.31E-12 | 2.14 | 7.36E-10 | 2.29 | 1.38E-11 | 2.5 | 3.75E-09 | 2.44 | 6.15E-15 |
| Lcn2 | Lipocalin 2 | | 2.21 | 3.36E-06 | 1.36 | >0.05 | 1.66 | 5.97E-03 | 2.66 | 1.49E-10 | 1.83 | 2.71E-0.4 |
| Cdkn1a | Cyclin-dependent kinase inhibitor 1A (P21) | | 1.62 | 6.04E-03 | 1.53 | 0.02 | 1.6 | 1.97E-03 | 1.7 | 6.13E-03 | 1.6 | 7.51E-04 |
| Jdp2 | Jun dimerization protein 2, transcript variant 1 | | 1.52 | >0.05 | 1.43 | >0.05 | 1.37 | >0.05 | 1.57 | 0.04 | 1.38 | >0.05 |
| Btg1 | B-cell translocation gene 1, anti-proliferative | | 1.51 | 0.03 | 1.41 | >0.05 | 1.54 | 5.02E-03 | 1.56 | 0.04 | 1.46 | 0.03 |
| Cited2 | Cbp/p300-interacting transactivator, with Glu/Asp-rich carboxy-terminal domain, 2 | | 1.39 | >0.05 | 1.34 | >0.05 | 1.48 | 0.02 | 1.56 | 0.04 | 1.48 | 0.02 |
| Sulfur metabolism | | |  |  |  |  |  |  |  |  |  |  |
| Glrx | Glutaredoxin | | 1.65 | 4.09E-04 | 1.48 | 0.02 | 1.6 | 1.43E-03 | 1.63 | 0.02 | 1.6 | 5.95E-04 |
| Glrx1 | Glutaredoxin | | 1.65 | 0.03 | 1.41 | >0.05 | 1.47 | >0.05 | 1.51 | >0.05 | 1.65 | 1.44E-03 |
| Cell adhesion | | |  |  |  |  |  |  |  |  |  |  |
| Icam1 | Intercellular adhesion molecule 1 | | 2.54 | 1.15E-12 | 2.52 | 1.27E-13 | 2.76 | 1.25E-17 | 3.0 | 1.94E-13 | 2.76 | 5.20E-18 |

| Gene symbol | | Description | 1119-3 | | C10 | | C29 | | D6 | | D7 | |
| --- | --- | --- | --- | --- | --- | --- | --- | --- | --- | --- | --- | --- |
|  |  |  | FC | P-value | FC | P-value | FC | P-value | FC | P-value | FC | P-value |
| Cell adhesion (*Continued*) | | |  |  |  |  |  |  |  |  |  |  |
| Itga5 | Integrin alpha 5 (fibronectin receptor alpha) | | 2.2 | 2.56E-07 | 2.01 | 8.99E-06 | 2.19 | 8.76E-09 | 2.45 | 7.71E-09 | 2.38 | 6.67E-12 |
| Arhgef3 | Rho guanine nucleotide exchange factor (GEF) 3 | | 2.08 | 1.36E-05 | 1.59 | 3.15E-02 | 1.68 | 4.59E-03 | 2.32 | 9.56E-08 | 1.75 | 1.27E-04 |
| Sdc4 | Syndecan 4 | | 1.66 | 0.02 | 1.46 | >0.05 | 1.69 | 3.15E-03 | 1.82 | 7.79E-04 | 1.74 | 1.38E-04 |
| Ptpn12 | Protein tyrosine phosphatase, non-receptor type 12 | | 1.52 | >0.05 | 1.39 | >0.05 | 1.54 | 0.04 | 1.51 | >0.05 | 1.46 | >0.05 |
| Intracellular protein traffic | | |  |  |  |  |  |  |  |  |  |  |
| Nfkbia | Nuclear factor of kappa light polypeptide gene enhancer in B-cells inhibitor, alpha | | 3.58 | 1.78E-22 | 3.34 | 2.00E-21 | 4.25 | 1.50E-36 | 4.46 | 2.12E-25 | 3.73 | 2.96E-29 |
| Ehd1 | EH-domain containing 1 | | 3.45 | 8.17E-17 | 2.83 | 1.43E-12 | 3.22 | 2.12E-17 | 4.01 | 1.02E-21 | 3.5 | 1.30E-24 |
| Adora2b | Adenosine A2b receptor | | 2.2 | 5.39E-07 | 1.86 | 3.14E-04 | 2.13 | 1.24E-07 | 2.47 | 5.34E-09 | 2.08 | 3.05E-08 |
| Arhgef3 | Rho guanine nucleotide exchange factor (GEF) 3 | | 2.08 | 1.36E-05 | 1.59 | 3.15E-02 | 1.68 | 4.59E-03 | 2.32 | 9.56E-08 | 1.75 | 1.27E-04 |
| Stx11 | Syntaxin 11 (Stx11), transcript variant 1 | | 1.81 | 2.26E-03 | 1.57 | 0.04 | 1.89 | 1.03E-04 | 1.97 | 6.50E-05 | 1.86 | 4.98E-05 |
| Fnbp1l | Formin binding protein 1-like | | 1.72 | 4.46E-04 | 1.58 | 4.57E-03 | 1.68 | 2.45E-04 | 1.88 | 2.99E-04 | 1.82 | 4.90E-06 |
| Adrb2 | Adrenergic receptor, beta 2 | | 1.37 | >0.05 | 1.4 | >0.05 | 1.53 | 0.04 | 1.44 | >0.05 | 1.42 | >0.05 |
| Neuronal activities | | |  |  |  |  |  |  |  |  |  |  |
| Ehd1 | EH-domain containing 1 | | 3.45 | 8.17E-17 | 2.83 | 1.43E-12 | 3.22 | 2.12E-17 | 4.01 | 1.02E-21 | 3.5 | 1.30E-24 |
| Adora2b | Adenosine A2b receptor | | 2.2 | 5.39E-07 | 1.86 | 3.14E-04 | 2.13 | 1.24E-07 | 2.47 | 5.34E-09 | 2.08 | 3.05E-08 |
| Atf3 | Activating transcription factor 3 | | 2.19 | 1.00E-08 | 1.88 | 5.14E-06 | 2.21 | 8.81E-11 | 2.25 | 3.52E-07 | 2.1 | 3.03E-09 |
| Adrb2 | Adrenergic receptor, beta 2 | | 1.37 | >0.05 | 1.4 | >0.05 | 1.53 | 0.04 | 1.44 | >0.05 | 1.42 | >0.05 |
| Protein targeting and localization | | |  |  |  |  |  |  |  |  |  |  |
| Stx11 | Syntaxin 11 (Stx11), transcript variant 1 | | 1.81 | 2.26E-03 | 1.57 | 0.04 | 1.89 | 1.03E-04 | 1.97 | 6.50E-05 | 1.86 | 4.98E-05 |

| Gene symbol | | Description | 1119-3 | | C10 | | C29 | | D6 | | D7 | |
| --- | --- | --- | --- | --- | --- | --- | --- | --- | --- | --- | --- | --- |
|  |  |  | FC | P-value | FC | P-value | FC | P-value | FC | P-value | FC | P-value |
| Cell adhesion (*Continued*) | | |  |  |  |  |  |  |  |  |  |  |
| Zyx | Zyxin | | 1.57 | 6.85E-03 | 1.42 | >0.05 | 1.46 | 0.03 | 1.45 | >0.05 | 1.54 | 3.41E-03 |
| Ptpn12 | Protein tyrosine phosphatase, non-receptor type 12 | | 1.52 | >0.05 | 1.39 | >0.05 | 1.54 | 0.04 | 1.51 | >0.05 | 1.46 | >0.05 |
| Protein metabolism and modification | | |  |  |  |  |  |  |  |  |  |  |
| LOC240672 | Similar to MAP-kinase phosphatase (cpg21) | | 3.15 | 5.43E-16 | 2.8 | 4.99E-13 | 3.66 | 9.59E-27 | 3.58 | 2.96E-18 | 3.37 | 7.41E-24 |
| Tnfaip3 | Tumor necrosis factor, alpha-induced protein 3 | | 3.08 | 1.23E-13 | 2.62 | 1.05E-10 | 3.81 | 3.06E-25 | 3.79 | 5.55E-20 | 3.25 | 9.55E-22 |
| Ibrdc3 | Ring finger protein 19B | | 2.29 | 1.17E-08 | 2.19 | 4.19E-08 | 2.36 | 1.28E-11 | 2.37 | 3.87E-08 | 2.36 | 6.92E-12 |
| Dusp2 | Dual specificity phosphatase 2 | | 2.11 | 3.43E-06 | 2.03 | 8.45E-06 | 2.77 | 4.06E-16 | 2.31 | 1.23E-07 | 2.26 | 1.96E-10 |
| 2310004  N11Rik | Serine/threonine kinase 40 (Stk40), transcript variant 2 | | 2.06 | 1.15E-06 | 1.71 | 1.67E-03 | 1.92 | 1.32E-06 | 2.16 | 1.76E-06 | 1.93 | 6.60E-07 |
| Ikbke | Inhibitor of kappaB kinase epsilon | | 1.95 | 2.49E-04 | 1.71 | 4.53E-03 | 1.87 | 1.79E-04 | 2.25 | 3.52E-07 | 1.99 | 9.90E-07 |
| Stk40 | Serine/threonine kinase 40 (Stk40) | | 1.94 | 3.12E-04 | 1.68 | 7.80E-03 | 1.7 | 3.36E-03 | 1.95 | 8.27E-05 | 1.79 | 8.52E-05 |
| Plk2 | Polo-like kinase 2 (Drosophila) | | 1.91 | 7.08E-06 | 1.6 | 3.47E-03 | 1.92 | 3.75E-07 | 1.89 | 2.36E-04 | 1.74 | 4.43E-05 |
| Plk3 | Polo-like kinase 3 (Drosophila) | | 1.86 | 6.47E-04 | 1.66 | 0.01 | 1.75 | 1.05E-03 | 1.83 | 7.63E-04 | 1.81 | 2.86E-05 |
| Sqstm1 | Sequestosome 1 | | 1.81 | 7.85E-06 | 1.72 | 6.01E-05 | 1.83 | 4.39E-06 | 1.94 | 1.11E-04 | 1.9 | 2.55E-07 |
| Dusp16 | Dual specificity phosphatase 16, transcript variant A1 | | 1.75 | 6.04E-03 | 1.56 | >0.05 | 1.85 | 2.48E-04 | 1.88 | 2.74E-04 | 1.72 | 2.88E-04 |
| Casp4 | Caspase 4, apoptosis-related cysteine peptidase | | 1.74 | 6.85E-03 | 1.52 | >0.05 | 1.65 | 8.57E-03 | 1.69 | 7.11E-03 | 1.59 | 5.44E-03 |
| Slpi | Secretory leukocyte peptidase inhibitor | | 1.73 | 4.70E-03 | 1.37 | >0.05 | 1.48 | >0.05 | 1.63 | 0.02 | 1.53 | 0.01 |
| St3gal6 | ST3 beta-galactoside alpha-2,3-sialyltransferase 6 | | 1.68 | 0.01 | 1.48 | >0.05 | 1.78 | 4.90E-04 | 1.64 | 0.02 | 1.61 | 2.45E-03 |
| Irak2 | Interleukin-1 receptor-associated kinase 2 | | 1.66 | 2.30E-03 | 1.49 | 0.03 | 1.56 | 3.41E-03 | 1.7 | 6.13-E03 | 1.65 | 5.53E-04 |

| Gene symbol | | Description | 1119-3 | | C10 | | C29 | | D6 | | D7 | |
| --- | --- | --- | --- | --- | --- | --- | --- | --- | --- | --- | --- | --- |
|  |  |  | FC | P-value | FC | P-value | FC | P-value | FC | P-value | FC | P-value |
| Protein metabolism and modification (*Continued*) | | |  |  |  |  |  |  |  |  |  |  |
| Denr | Density-regulated protein | | 1.64 | 0.03 | 1.48 | >0.05 | 1.46 | >0.05 | 1.73 | 4.16E-03 | 1.52 | 0.02 |
| Dusp4 | Dual specificity phosphatase 4 | | 1.61 | 4.93E-03 | 1.52 | 0.02 | 1.71 | 9.45E-05 | 1.63 | 0.02 | 1.6 | 1.30E-03 |
| Mapkapk2 | MAP kinase-activated protein kinase 2 | | 1.56 | 0.01 | 1.5 | 0.03 | 1.48 | 0.02 | 1.69 | 7.55E-03 | 1.7 | 1.27E-04 |
| Ptpn12 | Protein tyrosine phosphatase, non-receptor type 12 | | 1.52 | >0.05 | 1.39 | >0.05 | 1.54 | 0.04 | 1.51 | >0.05 | 1.46 | >0.05 |
| Tgm2 | Transglutaminase 2, C polypeptide | | 1.52 | >0.05 | 1.32 | >0.05 | 1.32 | >0.05 | 1.62 | 0.02 | 1.45 | 0.04 |
| Tor3a | Torsin family 3, member A | | 1.44 | >0.05 | 1.37 | >0.05 | 1.49 | >0.05 | 1.6 | 0.03 | 1.44 | >0.05 |
| Mak16 | MAK16 homolog (S. cerevisiae) | | 1.41 | >0.05 | 1.33 | >0.05 | 1.49 | 0.02 | 1.57 | 0.04 | 1.46 | 0.03 |
| Fubp1 | Far upstream element (FUSE) binding protein 1 | | 1.37 | >0.05 | 1.38 | >0.05 | 1.4 | >0.05 | 1.45 | >0.05 | 1.5 | 0.02 |
| Homeostasis | | |  |  |  |  |  |  |  |  |  |  |
| Edn1 | Endothelin 1 | | 3.53 | 2.85E-16 | 2.28 | 8.95E-08 | 2.49 | 5.74E-10 | 3.51 | 1.20E-17 | 2.84 | 1.34E-15 |
| Lipid, fatty acid and steroid metabolism | | |  |  |  |  |  |  |  |  |  |  |
| Agpat4 | 1-acylglycerol-3-phosphate O-acyltransferase 4 (lysophosphatidic acid acyltransferase, delta) | | 2.61 | 1.33E-10 | 2.22 | 2.64E-07 | 2.43 | 1.84E-10 | 2.63 | 2.80E-10 | 2.41 | 5.82E-12 |
| St3gal6 | ST3 beta-galactoside alpha-2,3-sialyltransferase 6 | | 1.68 | 0.01 | 1.48 | >0.05 | 1.78 | 4.90E-04 | 1.64 | 0.02 | 1.61 | 2.45E-03 |
| Pip5k1a | Phosphatidylinositol-4-phosphate 5-kinase, type 1 alpha | | 1.63 | 4.17E-03 | 1.39 | >0.05 | 1.52 | 0.01 | 1.35 | >0.05 | 1.28 | >0.05 |
| Gdpd1 | Glycerophosphodiester phosphodiesterase domain containing 1 | | 1.6 | 0.02 | 1.45 | >0.05 | 1.58 | 0.01 | 1.66 | 0.01 | 1.62 | 1.95E-03 |
| Scd1 | Stearoyl-Coenzyme A desaturase 1 | | 1.36 | >0.05 | 1.34 | >0.05 | 1.55 | 6.25E-03 | 1.46 | >0.05 | 1.43 | >0.05 |
| Transport | | |  |  |  |  |  |  |  |  |  |  |
| Slc15a3 | Solute carrier family 15, member 3 | | 2.68 | 4.49E-14 | 2.24 | 4.28E-10 | 2.37 | 8.40E-13 | 3.08 | 4.19E-14 | 2.79 | 4.90E-18 |
| Mcoln2 | Mucolipin 2 (Mcoln2), transcript variant 2 | | 2.52 | 1.25E-10 | 2.1 | 1.26E-06 | 2.16 | 1.98E-08 | 2.58 | 7.49E-10 | 2.31 | 5.16E-11 |

| Gene symbol | | Description | 1119-3 | | C10 | | C29 | | D6 | | D7 | |
| --- | --- | --- | --- | --- | --- | --- | --- | --- | --- | --- | --- | --- |
|  |  |  | FC | P-value | FC | P-value | FC | P-value | FC | P-value | FC | P-value |
| Transport (*Continued*) | | |  |  |  |  |  |  |  |  |  |  |
| Lcn2 | Lipocalin 2 | | 2.21 | 3.36E-06 | 1.36 | >0.05 | 1.66 | 5.97E-03 | 2.66 | 1.49E-10 | 1.83 | 2.71E-0.4 |
| Arhgef3 | Rho guanine nucleotide exchange factor (GEF) 3 | | 2.08 | 1.36E-05 | 1.59 | 3.15E-02 | 1.68 | 4.59E-03 | 2.32 | 9.56E-08 | 1.75 | 1.27E-04 |
| Slc31a2 | Solute carrier family 31, member 2 | | 1.9 | 5.80E-04 | 1.75 | 2.67E-03 | 1.74 | 1.69E-03 | 2.08 | 8.16E-06 | 1.7 | 4.27E-04 |
| Slc25a25 | Solute carrier family 25 (mitochondrial carrier, phosphate carrier), member 25, nuclear gene encoding mitochondrial protein | | 1.66 | 0.01 | 1.58 | 0.03 | 1.66 | 3.14E-03 | 1.78 | 1.74E-03 | 1.65 | 9.73E-04 |
| Slc2a1 | Solute carrier family 2 (facilitated glucose transporter), member 1 | | 1.47 | 0.03 | 1.4 | >0.05 | 1.44 | 0.04 | 1.52 | >0.05 | 1.54 | 3.30E-03 |
| Adrb2 | Adrenergic receptor, beta 2 | | 1.37 | >0.05 | 1.4 | >0.05 | 1.53 | 0.04 | 1.44 | >0.05 | 1.42 | >0.05 |
| Other metabolism | | |  |  |  |  |  |  |  |  |  |  |
| Adora2b | Adenosine A2b receptor | | 2.2 | 5.39E-07 | 1.86 | 3.14E-04 | 2.13 | 1.24E-07 | 2.47 | 5.34E-09 | 2.08 | 3.05E-08 |
| Ccrn4l | CCR4 carbon catabolite repression 4-like (S. cerevisiae) | | 1.91 | 3.10E-04 | 1.67 | 8.91E-03 | 2.1 | 5.94E-07 | 2.2 | 8.73E-07 | 2.14 | 7.90E-09 |
| Denr | Density-regulated protein | | 1.64 | 0.03 | 1.48 | >0.05 | 1.46 | >0.05 | 1.73 | 4.16E-03 | 1.52 | 0.02 |
| Gdpd1 | Glycerophosphodiester phosphodiesterase domain containing 1 | | 1.6 | 0.02 | 1.45 | >0.05 | 1.58 | 0.01 | 1.66 | 0.01 | 1.62 | 1.95E-03 |
| Nucleoside, nucleotide and nucleic acid metabolism | | |  |  |  |  |  |  |  |  |  |  |
| Nfkbiz | Nuclear factor of kappa light polypeptide gene enhancer in B-cells inhibitor, zeta | | 15.58 | 3.68E-118 | 11.51 | 1.42E-100 | 18.61 | 3.69E-151 | 17.88 | 3.63E-96 | 14.34 | 1.80E-128 |
| Nfkbia | Nuclear factor of kappa light polypeptide gene enhancer in B-cells inhibitor, alpha | | 3.58 | 1.78E-22 | 3.34 | 2.00E-21 | 4.25 | 1.50E-36 | 4.46 | 2.12E-25 | 3.73 | 2.96E-29 |
| Ets2 | E26 avian leukemia oncogene 2, 3' domain | | 2.38 | 1.31E-12 | 2.14 | 7.36E-10 | 2.29 | 1.38E-11 | 2.5 | 3.75E-09 | 2.44 | 6.15E-15 |
| Zfp36 | Zinc finger protein 36 | | 2.26 | 1.04E-09 | 2.09 | 1.49E-08 | 2.89 | 3.36E-19 | 2.48 | 3.75E-09 | 2.27 | 1.14E-11 |
| Adora2b | Adenosine A2b receptor | | 2.2 | 5.39E-07 | 1.86 | 3.14E-04 | 2.13 | 1.24E-07 | 2.47 | 5.34E-09 | 2.08 | 3.05E-08 |

| Gene symbol | | | | Description | 1119-3 | | | | C10 | | | | C29 | | | | D6 | | | | D7 | | |
| --- | --- | --- | --- | --- | --- | --- | --- | --- | --- | --- | --- | --- | --- | --- | --- | --- | --- | --- | --- | --- | --- | --- | --- |
|  |  |  |  |  | FC | | P-value | | FC | | P-value | | FC | | P-value | | FC | | P-value | | FC | | P-value |
| Nucleoside, nucleotide and nucleic acid metabolism (*Continued*) | | | | | | |  | |  | |  | |  | |  | |  | |  | |  | |  |
| Atf3 | | | Activating transcription factor 3 | | 2.19 | | 1.00E-08 | | 1.88 | | 5.14E-06 | | 2.21 | | 8.81E-11 | | 2.25 | | 3.52E-07 | | 2.1 | | 3.03E-09 |
| Pou2f2 | | | POU domain, class 2, transcription factor 2 | | 2.15 | | 8.50E-06 | | 1.94 | | 7.04E-05 | | 2.05 | | 4.91E-06 | | 2.38 | | 3.15E-08 | | 2.1 | | 6.71E-08 |
| Gadd45a | | | Growth arrest and DNA-damage-inducible 45 alpha | | 2.06 | | 4.13E-05 | | 1.86 | | 3.25E-04 | | 2.19 | | 3.43E-07 | | 2.47 | | 5.81E-09 | | 2.12 | | 2.93E-08 |
| Relb | | | Avian reticuloendotheliosis viral (v-rel) oncogene related B | | 1.94 | | 2.86E-04 | | 1.87 | | 2.68E-04 | | 1.94 | | 4.10E-05 | | 2.09 | | 7.15E-06 | | 2.01 | | 2.55E-07 |
| Plagl2 | | | Pleiomorphic adenoma gene-like 2 | | 1.93 | | 4.95E-06 | | 1.6 | | 3.76E-03 | | 1.8 | | 9.01E-06 | | 2.06 | | 1.25E-05 | | 1.84 | | 3.21E-06 |
| Bcl3 | | | B-cell leukemia/lymphoma 3 | | 1.65 | | 0.02 | | 1.65 | | 0.01 | | 1.86 | | 1.89E-04 | | 1.8 | | 1.21E-03 | | 1.72 | | 3.59E-04 |
| Dtx4 | | | Deltex 4 homolog (Drosophila) | | 1.65 | | 0.01 | | 1.62 | | 0.01 | | 1.65 | | 3.20E-03 | | 1.86 | | 3.93E-04 | | 1.82 | | 2.05E-05 |
| Znrf1 | | | Zinc and ring finger 1 | | 1.61 | | 0.04 | | 1.42 | | >0.05 | | 1.34 | | >0.05 | | 1.58 | | 0.04 | | 1.49 | | 0.03 |
| Jdp2 | | | Jun dimerization protein 2, transcript variant 1 | | 1.52 | | >0.05 | | 1.43 | | >0.05 | | 1.37 | | >0.05 | | 1.57 | | 0.04 | | 1.38 | | >0.05 |
| Egr1 | | | Early growth response 1 | | 1.51 | | 0.02 | | 1.56 | | 5.28E-03 | | 1.85 | | 3.29E-06 | | 1.59 | | 0.03 | | 1.6 | | 7.09E-04 |
| Rel | | | Reticuloendotheliosis oncogene | | 1.49 | | >0.05 | | 1.38 | | >0.05 | | 1.72 | | 2.60E-03 | | 1.66 | | 0.01 | | 1.51 | | 0.02 |
| Klf2 | | | Kruppel-like factor 2 (lung) | | 1.43 | | >0.05 | | 1.53 | | >0.05 | | 1.42 | | >0.05 | | 1.54 | | >0.05 | | 1.59 | | 2.90E-03 |
| Cited2 | | | Cbp/p300-interacting transactivator, with Glu/Asp-rich carboxy-terminal domain, 2 | | 1.39 | | >0.05 | | 1.34 | | >0.05 | | 1.48 | | 0.02 | | 1.56 | | 0.04 | | 1.48 | | 0.02 |
| Fubp1 | | | Far upstream element (FUSE) binding protein 1 | | 1.37 | | >0.05 | | 1.38 | | >0.05 | | 1.4 | | >0.05 | | 1.45 | | >0.05 | | 1.5 | | 0.02 |
| Egr2 | | | Early growth response 2 | | 1.31 | | >0.05 | | 1.34 | | >0.05 | | 1.67 | | 5.92E-03 | | 1.44 | | >0.05 | | 1.45 | | >0.05 |
| Sensory perception | | | | |  | |  | |  | |  | |  | |  | |  | |  | |  | |  |
| Adora2b | | | Adenosine A2b receptor | | 2.2 | | 5.39E-07 | | 1.86 | | 3.14E-04 | | 2.13 | | 1.24E-07 | | 2.47 | | 5.34E-09 | | 2.08 | | 3.05E-08 |
| Non-vertebrate process | | | | |  | |  | |  | |  | |  | |  | |  | |  | |  | |  |
| Irak2 | | | Interleukin-1 receptor-associated kinase 2 | | 1.66 | | 2.30E-03 | | 1.49 | | 0.03 | | 1.56 | | 3.41E-03 | | 1.7 | | 6.13-E03 | | 1.65 | | 5.53E-04 |
| Gene symbol | | | | Description | 1119-3 | | | | C10 | | | | C29 | | | | D6 | | | | D7 | | |
|  |  |  |  |  | FC | | P-value | | FC | | P-value | | FC | | P-value | | FC | | P-value | | FC | | P-value |
| Muscle contraction | | | | | | |  | |  | |  | |  | |  | |  | |  | |  | |  |
| Adora2b | | | Adenosine A2b receptor | | 2.2 | | 5.39E-07 | | 1.86 | | 3.14E-04 | | 2.13 | | 1.24E-07 | | 2.47 | | 5.34E-09 | | 2.08 | | 3.05E-08 |
| Electron transport | | | | |  | |  | |  | |  | |  | |  | |  | |  | |  | |  |
| Il4i1 | | | Interleukin 4 induced 1 | | 4.0 | | 3.19E-20 | | 3.11 | | 4.16E-15 | | 3.64 | | 2.06E-20 | | 4.56 | | 4.58E-26 | | 3.88 | | 8.18E-29 |
| Carbohydrate metabolism | | | | |  | |  | |  | |  | |  | |  | |  | |  | |  | |  |
| Slc2a1 | | | Solute carrier family 2 (facilitated glucose transporter), member 1 | | 1.47 | | 0.03 | | 1.4 | | >0.05 | | 1.44 | | 0.04 | | 1.52 | | >0.05 | | 1.54 | | 3.30E-03 |
| Blood circulation and gas exchange | | | | |  | |  | |  | |  | |  | |  | |  | |  | |  | |  |
| Edn1 | | | Endothelin 1 | | 3.53 | | 2.85E-16 | | 2.28 | | 8.95E-08 | | 2.49 | | 5.74E-10 | | 3.51 | | 1.20E-17 | | 2.84 | | 1.34E-15 |
| Amino acid metabolism | | | | |  | |  | |  | |  | |  | |  | |  | |  | |  | |  |
| Arhgef3 | | | Rho guanine nucleotide exchange factor (GEF) 3 | | 2.08 | | 1.36E-05 | | 1.59 | | 3.15E-02 | | 1.68 | | 4.59E-03 | | 2.32 | | 9.56E-08 | | 1.75 | | 1.27E-04 |
| Biological process unclassified | | | | |  | |  | |  | |  | |  | |  | |  | |  | |  | |  |
| Irg1 | | | Immunoresponsive gene 1 | | 12.29 | | 2.47E-99 | | 8.82 | | 9.26E-80 | | 10.29 | | 4.04E-96 | | 14.74 | | 1.45E-83 | | 11.29 | | 1.04E-106 |
| Traf1 | | | NOD-derived CD11c +ve dendritic cells cDNA, RIKEN full-length enriched library, clone:F630118K07 product:Tnf receptor-associated factor 1, full insert sequence | | 8.13 | | 5.31E-56 | | 5.68 | | 3.78E-37 | | 7.3 | | 1.67E-58 | | 9.22 | | 1.21E-56 | | 7.54 | | 1.42E-66 |
| Phlda1 | | | Pleckstrin homology-like domain, family A, member 1 | | 6.56 | | 3.88E-53 | | 5.04 | | 8.66E-43 | | 6.97 | | 4.86E-67 | | 7.27 | | 3.88E-45 | | 6.49 | | 9.56E-62 |
| Cd83 | | | CD83 antigen | | 4.01 | | 7.25E-26 | | 3.31 | | 4.48E-19 | | 4.32 | | 3.18E-36 | | 4.47 | | 1.92E-25 | | 3.94 | | 2.74E-31 |
| Zc3h12a | | | Zinc finger CCCH type containing 12A | | 3.1 | | 4.70E-13 | | 2.55 | | 4.28E-10 | | 3.25 | | 8.11E-17 | | 3.47 | | 2.23E-17 | | 2.93 | | 1.06E-17 |
| Ybx3 | | | 0 day neonate head cDNA, RIKEN full-length enriched library, clone:4833436O22 product:Y box protein 3, full insert sequence | | 2.88 | | 3.80E-14 | | 1.93 | | 7.17E-05 | | 2.21 | | 1.25E-08 | | 3.09 | | 4.01E-14 | | 2.47 | | 5.72E-13 |
| Tnip1 | | | TNFAIP3 interacting protein 1 | | 2.74 | | 3.26E-14 | | 2.41 | | 2.57E-11 | | 2.59 | | 2.74E-15 | | 3.03 | | 1.23E-13 | | 2.84 | | 3.16E-18 |
| 2310016C08Rik | | | RIKEN cDNA 2310016C08 gene | | 2.71 | | 2.82E-14 | | 2.29 | | 1.41E-10 | | 2.44 | | 1.27E-13 | | 2.7 | | 6.54E-11 | | 2.57 | | 4.21E-15 |
| Gene symbol | | Description | | | 1119-3 | | | C10 | | | | C29 | | | | D6 | | | | D7 | | | |
|  |  |  |  |  | FC | P-value | | FC | | P-value | | FC | | P-value | | FC | | P-value | | FC | | P-value | |
| Biological process unclassified (*Continued*) | | | | |  |  | |  | |  | |  | |  | |  | |  | |  | |  | |
| Slc11a2 | 9 days embryo whole body cDNA, RIKEN full-length enriched library, clone:D030025E18 product:solute carrier family 11 (proton-coupled divalent metal ion transporters), member 2, full insert sequence | | | | 2.67 | 2.30E-12 | | 2.18 | | 1.79E-07 | | 2.34 | | 8.81E-11 | | 2.79 | | 1.07E-11 | | 2.51 | | 1.55E-13 | |
| Osm | Oncostatin M | | | | 2.33 | 1.93E-09 | | 1.77 | | 5.49E-04 | | 2.21 | | 3.89E-10 | | 2.4 | | 2.27E-08 | | 2.11 | | 5.78E-09 | |
| AI850995 | Expressed sequence AI850995 | | | | 2.26 | 7.60E-08 | | 2.02 | | 7.82E-06 | | 2.07 | | 2.91E-07 | | 2.33 | | 7.14E-08 | | 2.41 | | 2.75E-12 | |
| Tnfsf9 | Tumor necrosis factor (ligand) superfamily, member 9 | | | | 2.21 | 1.17E-06 | | 1.99 | | 2.84E-05 | | 2.62 | | 1.22E-12 | | 2.53 | | 1.80E-09 | | 2.22 | | 9.36E-10 | |
| Rapgef2 | Rap guanine nucleotide exchange factor (GEF) 2 | | | | 2.13 | 8.38E-06 | | 1.96 | | 5.46E-05 | | 2.31 | | 1.14E-08 | | 2.29 | | 1.66E-07 | | 2.27 | | 2.81E-10 | |
| A430093F15Rik | RIKEN cDNA A430093F15 gene | | | | 2.1 | 1.95E-05 | | 1.77 | | 1.71E-03 | | 1.91 | | 7.67E-05 | | 2.3 | | 1.43E-07 | | 2.08 | | 1.45E-07 | |
| Rsad2 | Radical S-adenosyl methionine domain containing 2 | | | | 2.08 | 2.75E-05 | | 1.9 | | 1.65E-04 | | 2.06 | | 4.39E-06 | | 2.24 | | 3.74E-07 | | 2.02 | | 2.03E-07 | |
| Spata13 | EST X83327 | | | | 2.03 | 5.32E-07 | | 2.06 | | 5.03E-08 | | 2.2 | | 1.22E-10 | | 2.25 | | 3.68E-07 | | 2.0 | | 5.13E-08 | |
| Slc4a7 | Solute carrier family 4, sodium bicarbonate cotransporter, member 7 | | | | 1.98 | 8.38E-06 | | 1.69 | | 2.73E-03 | | 1.83 | | 1.89E-05 | | 1.69 | | 7.11E-03 | | 1.54 | | 8.34E-03 | |
| Nfkb1 | 0 day neonate kidney cDNA, RIKEN full-length enriched library, clone:D630036B16 product:nuclear factor of kappa light chain gene enhancer in B-cells 1, p105, full insert sequence | | | | 1.97 | 2.26E-06 | | 1.71 | | 3.14E-04 | | 1.78 | | 1.48E-05 | | 2.04 | | 1.82E-05 | | 1.85 | | 2.67E-06 | |
| Nfkbid | Nuclear factor of kappa light polypeptide gene enhancer in B cells inhibitor, delta | | | | 1.93 | 3.97E-06 | | 1.68 | | 5.72E-04 | | 2.15 | | 8.45E-10 | | 2.1 | | 5.53E-06 | | 1.88 | | 1.06E-06 | |
| Slc7a11 | 16 days neonate thymus cDNA, RIKEN full-length enriched library, clone:A130021E01 product:solute carrier family 7 (cationic amino acid transporter, y+ system), member 11, full insert sequence | | | | 1.93 | 3.04E-04 | | 1.71 | | 5.21E-03 | | 1.74 | | 1.85E-03 | | 1.9 | | 2.12E-04 | | 1.9 | | 3.21E-06 | |

| Gene symbol | | Description | 1119-3 | | C10 | | C29 | | D6 | | D7 | |
| --- | --- | --- | --- | --- | --- | --- | --- | --- | --- | --- | --- | --- |
|  |  |  | FC | P-value | FC | P-value | FC | P-value | FC | P-value | FC | P-value |
| Biological process unclassified (*Continued*) | | |  |  |  |  |  |  |  |  |  |  |
| Herpud1 | Homocysteine-inducible, endoplasmic reticulum stress-inducible, ubiquitin-like domain member 1 | | 1.85 | 8.38E-06 | 1.66 | 6.09E-04 | 1.85 | 2.96E-06 | 1.94 | 1.12E-04 | 1.8 | 4.37E-06 |
| LOC212399 | Similar to actin, gamma, cytoplasmic | | 1.8 | 2.45E-03 | 1.69 | 6.80E-03 | 1.8 | 5.09E-04 | 1.89 | 2.40E-04 | 1.93 | 1.61E-06 |
| Nfkbie | Nuclear factor of kappa light polypeptide gene enhancer in B cells inhibitor, epsilon | | 1.71 | 0.01 | 1.71 | 4.57E-03 | 1.87 | 1.79E-04 | 1.88 | 2.76E-04 | 1.78 | 1.27E-04 |
| Odc1 | Ornithine decarboxylase, structural 1 | | 1.7 | 9.60E-05 | 1.54 | 4.73E-03 | 1.69 | 1.75E-04 | 1.76 | 5.60E-05 | 1.63 | 4.94E-04 |
| Plekho2 | Pleckstrin homology domain containing, family O member 2 | | 1.69 | 4.46E-04 | 1.65 | 8.36E-04 | 1.78 | 1.98E-05 | 1.81 | 9.47E-04 | 1.91 | 1.46E-07 |
| Pim3 | Proviral integration site 3 | | 1.69 | 3.13E-03 | 1.57 | 0.03 | 1.62 | 2.68E-03 | 1.7 | 6.24E-03 | 1.61 | 1.77E-03 |
| LOC100047963 | Similar to ADIR1 | | 1.69 | 6.63E-03 | 1.66 | 9.10E-03 | 1.66 | 3.00E-03 | 1.71 | 5.09E-03 | 1.77 | 7.06E-05 |
| 4930431B09Rik | RIKEN cDNA 4930431B09 gene | | 1.67 | 0.01 | 1.44 | >0.05 | 1.71 | 2.10E-03 | 1.62 | 0.02 | 1.49 | 0.02 |
| LOC100048376 | Similar to CAP-GLY domain containing linker protein family, member 4, transcript variant 1 | | 1.66 | 0.02 | 1.46 | >0.05 | 1.42 | >0.05 | 1.56 | >0.05 | 1.48 | >0.05 |
| 1200016E24Rik | RIKEN cDNA 1200016E24 gene | | 1.66 | 0.02 | 1.35 | >0.05 | 1.33 | >0.05 | 1.6 | 0.03 | 1.57 | 0.02 |
| Mfsd2 | Major facilitator superfamily domain containing 2 | | 1.65 | 0.02 | 1.51 | >0.05 | 1.6 | 0.02 | 1.77 | 2.07E-03 | 1.69 | 4.97E-04 |
| Fbxo33 | F-box protein 33 | | 1.63 | 0.03 | 1.64 | 0.02 | 1.58 | 0.02 | 1.59 | 0.03 | 1.63 | 1.57E-03 |
| Dnmt3l | DNA (cytosine-5-)-methyltransferase 3-like (Dnmt3l), transcript variant 2 | | 1.63 | 0.03 | 1.29 | >0.05 | 1.3 | >0.05 | 1.7 | 6.13E-03 | 1.45 | 0.04 |
| Rn18s | 18S ribosomal RNA | | 1.6 | 0.03 | 1.47 | >0.05 | 1.66 | 2.85E-03 | 1.29 | >0.05 | 1.06 | >0.05 |
| LOC100038882 | Hypothetical protein LOC100038882 | | 1.6 | 0.04 | 1.49 | >0.05 | 1.58 | 0.02 | 1.7 | 6.31E-03 | 1.53 | 0.02 |

| Gene symbol | | Description | 1119-3 | | C10 | | C29 | | D6 | | D7 | |
| --- | --- | --- | --- | --- | --- | --- | --- | --- | --- | --- | --- | --- |
|  |  |  | FC | P-value | FC | P-value | FC | P-value | FC | P-value | FC | P-value |
| Biological process unclassified (*Continued*) | | |  |  |  |  |  |  |  |  |  |  |
| Yrdc | yrdC domain containing (E.coli) | | 1.56 | 0.02 | 1.46 | >0.05 | 1.52 | 9.48E-03 | 1.67 | 0.01 | 1.55 | 5.15E-03 |
| MALT-1 | 9.5 days embryo parthenogenote cDNA, RIKEN full-length enriched library, clone:B130046C19 product:similar to mucosa associated lymphoid tissue lymphoma translocation gene 1, full insert sequence | | 1.56 | >0.05 | 1.36 | >0.05 | 1.6 | 0.02 | 1.57 | 0.04 | 1.47 | >0.05 |
| A430084P05Rik | RIKEN cDNA A430084P05 gene | | 1.56 | >0.05 | 1.38 | >0.05 | 1.45 | >0.05 | 1.56 | 0.04 | 1.45 | >0.05 |
| Akna | AT-hook transcription factor | | 1.56 | >0.05 | 1.31 | >0.05 | 1.49 | >0.05 | 1.62 | 0.02 | 1.47 | 0.04 |
| Tm7sf4 | Dentrocyte expressed seven transmembrane protein | | 1.54 | >0.05 | 1.58 | 0.03 | 1.55 | 0.01 | 1.54 | >0.05 | 1.7 | 2.91E-04 |
| Zswim4 | Zinc finger, SWIM domain containing 4 | | 1.54 | >0.05 | 1.5 | >0.05 | 1.69 | 4.05E-03 | 1.55 | >0.05 | 1.65 | 1.11E-03 |
| Srgn | Serglycin | | 1.52 | >0.05 | 1.27 | >0.05 | 1.39 | >0.05 | 1.53 | >0.05 | 1.54 | 0.01 |
| 6330409N04Rik | RIKEN cDNA 6330409N04 gene | | 1.52 | >0.05 | 1.39 | >0.05 | 1.46 | >0.05 | 1.57 | 0.04 | 1.49 | 0.04 |
| Cd52 | CD52 antigen | | 1.52 | >0.05 | 1.4 | >0.05 | 1.4 | >0.05 | 1.59 | 0.03 | 1.55 | 5.91E-03 |
| LOC677448 | Similar to actin | | 1.48 | >0.05 | 1.5 | >0.05 | 1.39 | >0.05 | 1.61 | 0.03 | 1.51 | 0.02 |
| LOC278105 | Similar to Odc protein | | 1.48 | >0.05 | 1.31 | >0.05 | 1.47 | >0.05 | 1.74 | 3.46E-03 | 1.67 | 4.45E-03 |
| Gp49a | B6-derived CD11 +ve dendritic cells cDNA, RIKEN full-length enriched library, clone:F730015K05 product:glycoprotein 49 A, full insert sequence | | 1.44 | >0.05 | 1.36 | >0.05 | 1.45 | 0.03 | 1.58 | 0.04 | 1.58 | 1.95E-03 |
| Plek | Pleckstrin | | 1.42 | >0.05 | 1.32 | >0.05 | 1.6 | 1.97E-03 | 1.79 | 1.41E-03 | 1.78 | 2.25E-05 |
| LOC100048346 | Similar to ubiquitin specific protease UBP43 | | 1.38 | >0.05 | 1.46 | >0.05 | 1.51 | 0.03 | 1.47 | >0.05 | 1.47 | 0.03 |
| Arc | Activity regulated cytoskeletal-associated protein | | 1.37 | >0.05 | 1.36 | >0.05 | 1.62 | 0.01 | 1.42 | >0.05 | 1.44 | >0.05 |
| Zfp330 | Zinc finger protein 330 | | 1.34 | >0.05 | 1.35 | >0.05 | 1.45 | >0.05 | 1.58 | 0.04 | 1.52 | 0.01 |
