# Supplementary material for: Early transcriptional responses of internalization defective Brucella abortus mutants in professional phagocytes, RAW 264.7
Source: BMC Genomics. 2013 Jun 27;14:426. doi: 10.1186/1471-2164-14-426 (PMC3716731; doi:10.1186/1471-2164-14-426)
Supplement: Additional file 2 — Genes with down-regulated in RAW 264.7 infected with each B.abortus compare to uninfected macrophage. [file 1471-2164-14-426-S2.docx]

Additional file 2. Genes with down-regulated in RAW 264.7 infected with each *B.abortus* compare to uninfected macrophage.

| Gene symbol | | Description | 1119-3 | | C10 | | C29 | | D6 | | D7 | |
| --- | --- | --- | --- | --- | --- | --- | --- | --- | --- | --- | --- | --- |
|  |  |  | FC | P-value | FC | P-value | FC | P-value | FC | P-value | FC | P-value |
| Signal transduction | | |  |  |  |  |  |  |  |  |  |  |
| Cxcr4 | Chemokine (C-X-C motif) receptor 4 | | -2.17 | 6.43E-06 | -1.81 | 8.36E-04 | -2.13 | 9.94E-07 | -2.63 | 2.59E-10 | -2.12 | 4.46E-07 |
| 5430435G22Rik | RIKEN cDNA 5430435G22 gene | | -1.81 | 2.26E-03 | -1.57 | 0.04 | -1.74 | 1.90E-03 | -1.93 | 1.25E-04 | -1.68 | 5.34E-04 |
| Tspan14 | Tetraspanin 14 | | -1.73 | 1.60E-03 | -1.43 | >0.05 | -1.45 | 0.04 | -1.72 | 4.53E-03 | -1.52 | 0.01 |
| Fblim1 | Filamin binding LIM protein 1 | | -1.72 | 2.29E-03 | -1.53 | 0.04 | -1.49 | 0.02 | -1.61 | 0.02 | -1.56 | 5.47E-03 |
| Gadd45g | Growth arrest and DNA-damage-inducible 45 gamma | | -1.67 | 0.01 | -1.57 | 0.04 | -1.71 | 2.79E-03 | -2.02 | 2.44E-05 | -1.68 | 8.07E-04 |
| Ebi2 | Epstein-Barr virus induced gene 2 | | -1.66 | 2.44E-03 | -1.56 | 0.01 | -1.64 | 5.84E-04 | -1.82 | 7.79E-04 | -1.69 | 2.25E-04 |
| Rab40c | Rab40c, member RAS oncogene family | | -1.53 | 0.03 | -1.38 | >0.05 | -1.62 | 1.53E-03 | -1.6 | 0.03 | -1.59 | 2.37E-03 |
| Dm15 | Dystrophia myotonica-protein kinase (Dmpk), transcript variant 1 | | -1.53 | >0.05 | -1.45 | >0.05 | -1.5 | >0.05 | -1.5 | >0.05 | -1.5 | 0.02 |
| Aatk | Apoptosis-associated tyrosine kinase | | -1.53 | >0.05 | -1.47 | >0.05 | -1.58 | 0.02 | -1.6 | 0.03 | -1.58 | 4.43E-03 |
| Tlk1 | Tousled-like kinase 1 | | -1.49 | 0.04 | -1.39 | >0.05 | -1.43 | >0.05 | -1.55 | >0.05 | -1.52 | 8.56E-03 |
| Dgkg | Diacylglycerol kinase, gamma | | -1.46 | >0.05 | -1.41 | >0.05 | -1.45 | >0.05 | -1.52 | >0.05 | -1.59 | 0.02 |
| E2f2 | E2F transcription factor 2 | | -1.45 | >0.05 | -1.37 | >0.05 | -1.38 | >0.05 | -1.56 | 0.04 | -1.52 | 0.04 |
| Rab27a | RAB27A, member RAS oncogene family | | -1.44 | >0.05 | -1.36 | >0.05 | -1.37 | >0.05 | -1.57 | 0.04 | -1.5 | 0.02 |
| Il6ra | Interleukin 6 receptor, alpha | | -1.44 | >0.05 | -1.32 | >0.05 | -1.38 | >0.05 | -1.53 | >0.05 | -1.53 | 0.04 |
| LOC670044 | Similar to Mothers against decapentaplegic homolog 6 (SMAD 6) (Mothers against DPP homolog 6) (Smad6) (Mad homolog 7) | | -1.44 | >0.05 | -1.35 | >0.05 | -1.48 | >0.05 | -1.57 | 0.04 | -1.45 | >0.05 |
| Tec | Cytoplasmic tyrosine kinase, Dscr28C related (Drosophila) | | -1.37 | >0.05 | -1.29 | >0.05 | -1.39 | >0.05 | -1.61 | 0.02 | -1.46 | 0.04 |

| Gene symbol | | Description | 1119-3 | | C10 | | C29 | | D6 | | D7 | |
| --- | --- | --- | --- | --- | --- | --- | --- | --- | --- | --- | --- | --- |
|  |  |  | FC | P-value | FC | P-value | FC | P-value | FC | P-value | FC | P-value |
| Signal transduction (*Continued*) | | |  |  |  |  |  |  |  |  |  |  |
| Nrp1 | Neuropilin 1 | | -1.35 | >0.05 | -1.33 | >0.05 | -1.32 | >0.05 | -1.58 | 0.04 | -1.47 | 0.03 |
| Immunity and defense | | |  |  |  |  |  |  |  |  |  |  |
| Cxcr4 | Chemokine (C-X-C motif) receptor 4 | | -2.17 | 6.43E-06 | -1.81 | 8.36E-04 | -2.13 | 9.94E-07 | -2.63 | 2.59E-10 | -2.12 | 4.46E-07 |
| Gadd45g | Growth arrest and DNA-damage-inducible 45 gamma | | -1.67 | 0.01 | -1.57 | 0.04 | -1.71 | 2.79E-03 | -2.02 | 2.44E-05 | -1.68 | 8.07E-04 |
| Il6ra | Interleukin 6 receptor, alpha | | -1.44 | >0.05 | -1.32 | >0.05 | -1.38 | >0.05 | -1.53 | >0.05 | -1.53 | 0.04 |
| LOC670044 | Similar to Mothers against decapentaplegic homolog 6 (SMAD 6) (Mothers against DPP homolog 6) (Smad6) (Mad homolog 7) | | -1.44 | >0.05 | -1.35 | >0.05 | -1.48 | >0.05 | -1.57 | 0.04 | -1.45 | >0.05 |
| Tec | Cytoplasmic tyrosine kinase, Dscr28C related (Drosophila) | | -1.37 | >0.05 | -1.29 | >0.05 | -1.39 | >0.05 | -1.61 | 0.02 | -1.46 | 0.04 |
| Apoptosis | | |  |  |  |  |  |  |  |  |  |  |
| Cxcr4 | Chemokine (C-X-C motif) receptor 4 | | -2.17 | 6.43E-06 | -1.81 | 8.36E-04 | -2.13 | 9.94E-07 | -2.63 | 2.59E-10 | -2.12 | 4.46E-07 |
| Gadd45g | Growth arrest and DNA-damage-inducible 45 gamma | | -1.67 | 0.01 | -1.57 | 0.04 | -1.71 | 2.79E-03 | -2.02 | 2.44E-05 | -1.68 | 8.07E-04 |
| Aatk | Apoptosis-associated tyrosine kinase | | -1.53 | >0.05 | -1.47 | >0.05 | -1.58 | 0.02 | -1.6 | 0.03 | -1.58 | 4.43E-03 |
| Mybl2 | Myeloblastosis oncogene-like 2 | | -1.48 | >0.05 | -1.42 | >0.05 | -1.42 | >0.05 | -1.51 | >0.05 | -1.51 | 0.02 |
| Emp1 | Epithelial membrane protein 1 | | -1.44 | >0.05 | -1.4 | >0.05 | -1.44 | 0.04 | -1.57 | 0.04 | -1.47 | 0.02 |
| Tec | Cytoplasmic tyrosine kinase, Dscr28C related (Drosophila) | | -1.37 | >0.05 | -1.29 | >0.05 | -1.39 | >0.05 | -1.61 | 0.02 | -1.46 | 0.04 |
| Cell proliferation and differentiation | | |  |  |  |  |  |  |  |  |  |  |
| Fblim1 | Filamin binding LIM protein 1 | | -1.72 | 2.29E-03 | -1.53 | 0.04 | -1.49 | 0.02 | -1.61 | 0.02 | -1.56 | 5.47E-03 |
| Gadd45g | Growth arrest and DNA-damage-inducible 45 gamma | | -1.67 | 0.01 | -1.57 | 0.04 | -1.71 | 2.79E-03 | -2.02 | 2.44E-05 | -1.68 | 8.07E-04 |
| Mybl2 | Myeloblastosis oncogene-like 2 | | -1.48 | >0.05 | -1.42 | >0.05 | -1.42 | >0.05 | -1.51 | >0.05 | -1.51 | 0.02 |
| E2f2 | E2F transcription factor 2 | | -1.45 | >0.05 | -1.37 | >0.05 | -1.38 | >0.05 | -1.56 | 0.04 | -1.52 | 0.04 |
| Emp1 | Epithelial membrane protein 1 | | -1.44 | >0.05 | -1.4 | >0.05 | -1.44 | 0.04 | -1.57 | 0.04 | -1.47 | 0.02 |
| Il6ra | Interleukin 6 receptor, alpha | | -1.44 | >0.05 | -1.32 | >0.05 | -1.38 | >0.05 | -1.53 | >0.05 | -1.53 | 0.04 |

| Gene symbol | | Description | 1119-3 | | C10 | | C29 | | D6 | | D7 | |
| --- | --- | --- | --- | --- | --- | --- | --- | --- | --- | --- | --- | --- |
|  |  |  | FC | P-value | FC | P-value | FC | P-value | FC | P-value | FC | P-value |
| Developmental processes | | |  |  |  |  |  |  |  |  |  |  |
| Cxcr4 | Chemokine (C-X-C motif) receptor 4 | | -2.17 | 6.43E-06 | -1.81 | 8.36E-04 | -2.13 | 9.94E-07 | -2.63 | 2.59E-10 | -2.12 | 4.46E-07 |
| Enc1 | Ectodermal-neural cortex 1 | | -2.01 | 1.01E-04 | -1.77 | 1.71E-03 | -1.9 | 1.03E-04 | -1.98 | 5.11E-05 | -1.98 | 9.61E-07 |
| E2f2 | E2F transcription factor 2 | | -1.45 | >0.05 | -1.37 | >0.05 | -1.38 | >0.05 | -1.56 | 0.04 | -1.52 | 0.04 |
| Emp1 | Epithelial membrane protein 1 | | -1.44 | >0.05 | -1.4 | >0.05 | -1.44 | 0.04 | -1.57 | 0.04 | -1.47 | 0.02 |
| LOC670044 | Similar to Mothers against decapentaplegic homolog 6 (SMAD 6) (Mothers against DPP homolog 6) (Smad6) (Mad homolog 7) | | -1.44 | >0.05 | -1.35 | >0.05 | -1.48 | >0.05 | -1.57 | 0.04 | -1.45 | >0.05 |
| Nrp1 | Neuropilin 1 | | -1.35 | >0.05 | -1.33 | >0.05 | -1.32 | >0.05 | -1.58 | 0.04 | -1.47 | 0.03 |
| Cell cycle | | |  |  |  |  |  |  |  |  |  |  |
| Gadd45g | Growth arrest and DNA-damage-inducible 45 gamma | | -1.67 | 0.01 | -1.57 | 0.04 | -1.71 | 2.79E-03 | -2.02 | 2.44E-05 | -1.68 | 8.07E-04 |
| Esco2 | Establishment of cohesion 1 homolog 2 (S. cerevisiae) | | -1.53 | >0.05 | -1.46 | >0.05 | -1.47 | >0.05 | -1.66 | 0.01 | -1.58 | 4.47E-03 |
| 2900026A02Rik | RIKEN cDNA 2900026A02 gene | | -1.53 | >0.05 | -1.36 | >0.05 | -1.46 | >0.05 | -1.56 | 0.04 | -1.46 | >0.05 |
| Tlk1 | Tousled-like kinase 1 | | -1.49 | 0.04 | -1.39 | >0.05 | -1.43 | >0.05 | -1.55 | >0.05 | -1.52 | 8.56E-03 |
| Mybl2 | Myeloblastosis oncogene-like 2 | | -1.48 | >0.05 | -1.42 | >0.05 | -1.42 | >0.05 | -1.51 | >0.05 | -1.51 | 0.02 |
| E2f2 | E2F transcription factor 2 | | -1.45 | >0.05 | -1.37 | >0.05 | -1.38 | >0.05 | -1.56 | 0.04 | -1.52 | 0.04 |
| Emp1 | Epithelial membrane protein 1 | | -1.44 | >0.05 | -1.4 | >0.05 | -1.44 | 0.04 | -1.57 | 0.04 | -1.47 | 0.02 |
| Sesn1 | Sestrin 1 | | -1.43 | >0.05 | -1.3 | >0.05 | -1.38 | >0.05 | -1.56 | 0.04 | -1.46 | >0.05 |
| Cell structure and motility | | |  |  |  |  |  |  |  |  |  |  |
| Cxcr4 | Chemokine (C-X-C motif) receptor 4 | | -2.17 | 6.43E-06 | -1.81 | 8.36E-04 | -2.13 | 9.94E-07 | -2.63 | 2.59E-10 | -2.12 | 4.46E-07 |
| Enc1 | Ectodermal-neural cortex 1 | | -2.01 | 1.01E-04 | -1.77 | 1.71E-03 | -1.9 | 1.03E-04 | -1.98 | 5.11E-05 | -1.98 | 9.61E-07 |
| Fblim1 | Filamin binding LIM protein 1 | | -1.72 | 2.29E-03 | -1.53 | 0.04 | -1.49 | 0.02 | -1.61 | 0.02 | -1.56 | 5.47E-03 |
| Dm15 | Dystrophia myotonica-protein kinase (Dmpk), transcript variant 1 | | -1.53 | >0.05 | -1.45 | >0.05 | -1.5 | >0.05 | -1.5 | >0.05 | -1.5 | 0.02 |
| Klhl17 | Kelch-like 17 (Drosophila) | | -1.46 | >0.05 | -1.37 | >0.05 | -1.44 | >0.05 | -1.46 | >0.05 | -1.52 | 0.01 |

| Gene symbol | | Description | 1119-3 | | C10 | | C29 | | D6 | | D7 | |
| --- | --- | --- | --- | --- | --- | --- | --- | --- | --- | --- | --- | --- |
|  |  |  | FC | P-value | FC | P-value | FC | P-value | FC | P-value | FC | P-value |
| Oncogenesis | | |  |  |  |  |  |  |  |  |  |  |
| Fblim1 | Filamin binding LIM protein 1 | | -1.72 | 2.29E-03 | -1.53 | 0.04 | -1.49 | 0.02 | -1.61 | 0.02 | -1.56 | 5.47E-03 |
| Phf17 | PHD finger protein 17 | | -1.7 | 0.01 | -1.59 | 0.03 | -1.62 | 0.01 | -1.72 | 4.73E-03 | -1.55 | 8.58E-03 |
| Emp1 | Epithelial membrane protein 1 | | -1.44 | >0.05 | -1.4 | >0.05 | -1.44 | 0.04 | -1.57 | 0.04 | -1.47 | 0.02 |
| LOC670044 | Similar to Mothers against decapentaplegic homolog 6 (SMAD 6) (Mothers against DPP homolog 6) (Smad6) (Mad homolog 7) | | -1.44 | >0.05 | -1.35 | >0.05 | -1.48 | >0.05 | -1.57 | 0.04 | -1.45 | >0.05 |
| Sulfur metabolism | | |  |  |  |  |  |  |  |  |  |  |
| Gclm | Glutamate-cysteine ligase, modifier subunit | | -1.46 | >0.05 | -1.44 | >0.05 | -1.51 | 0.01 | -1.46 | >0.05 | -1.37 | >0.05 |
| Mgst2 | Microsomal glutathione S-transferase 2 | | -1.41 | >0.05 | -1.42 | >0.05 | -1.46 | >0.05 | -1.44 | >0.05 | -1.52 | 0.02 |
| Cell adhesion | | |  |  |  |  |  |  |  |  |  |  |
| Tspan14 | Tetraspanin 14 | | -1.73 | 1.60E-03 | -1.43 | >0.05 | -1.45 | 0.04 | -1.72 | 4.53E-03 | -1.52 | 0.01 |
| Intracellular protein traffic | | |  |  |  |  |  |  |  |  |  |  |
| 5430435G22Rik | RIKEN cDNA 5430435G22 gene | | -1.81 | 2.26E-03 | -1.57 | 0.04 | -1.74 | 1.90E-03 | -1.93 | 1.25E-04 | -1.68 | 5.34E-04 |
| Rin2 | Ras and Rab interactor 2 | | -1.59 | >0.05 | -1.42 | >0.05 | -1.54 | 0.04 | -1.55 | >0.05 | -1.55 | 0.01 |
| Rab40c | Rab40c, member RAS oncogene family | | -1.53 | 0.03 | -1.38 | >0.05 | -1.62 | 1.53E-03 | -1.6 | 0.03 | -1.59 | 2.37E-03 |
| Rab27a | RAB27A, member RAS oncogene family | | -1.44 | >0.05 | -1.36 | >0.05 | -1.37 | >0.05 | -1.57 | 0.04 | -1.5 | 0.02 |
| Snx30 | Sorting nexin family member 30 | | -1.39 | >0.05 | -1.28 | >0.05 | -1.32 | >0.05 | -1.58 | 0.04 | -1.45 | >0.05 |
| Neuronal activities | | |  |  |  |  |  |  |  |  |  |  |
| Klhl17 | Kelch-like 17 (Drosophila) | | -1.46 | >0.05 | -1.37 | >0.05 | -1.44 | >0.05 | -1.46 | >0.05 | -1.52 | 0.01 |
| Protein metabolism and modification | | |  |  |  |  |  |  |  |  |  |  |
| Dm15 | Dystrophia myotonica-protein kinase (Dmpk), transcript variant 1 | | -1.53 | >0.05 | -1.45 | >0.05 | -1.5 | >0.05 | -1.5 | >0.05 | -1.5 | 0.02 |
| Aatk | Apoptosis-associated tyrosine kinase | | -1.53 | >0.05 | -1.47 | >0.05 | -1.58 | 0.02 | -1.6 | 0.03 | -1.58 | 4.43E-03 |
| Tlk1 | Tousled-like kinase 1 | | -1.49 | 0.04 | -1.39 | >0.05 | -1.43 | >0.05 | -1.55 | >0.05 | -1.52 | 8.56E-03 |

| Gene symbol | | Description | 1119-3 | | C10 | | C29 | | D6 | | D7 | |
| --- | --- | --- | --- | --- | --- | --- | --- | --- | --- | --- | --- | --- |
|  |  |  | FC | P-value | FC | P-value | FC | P-value | FC | P-value | FC | P-value |
| Protein metabolism and modification *(Continued)* | | |  |  |  |  |  |  |  |  |  |  |
| Gcnt1 | Glucosaminyl (N-acetyl) transferase 1, core 2 | | -1.46 | >0.05 | -1.34 | >0.05 | -1.52 | >0.05 | -1.57 | 0.04 | -1.47 | 0.04 |
| Map3k1 | Mitogen-activated protein kinase kinase kinase 1 | | -1.46 | >0.05 | -1.45 | >0.05 | -1.56 | 5.60E-03 | -1.84 | 6.18E-04 | -1.65 | 7.51E-04 |
| Gclm | Glutamate-cysteine ligase, modifier subunit | | -1.46 | >0.05 | -1.44 | >0.05 | -1.51 | 0.01 | -1.46 | >0.05 | -1.37 | >0.05 |
| Mgst2 | Microsomal glutathione S-transferase 2 | | -1.41 | >0.05 | -1.42 | >0.05 | -1.46 | >0.05 | -1.44 | >0.05 | -1.52 | 0.02 |
| Tec | Cytoplasmic tyrosine kinase, Dscr28C related (Drosophila) | | -1.37 | >0.05 | -1.29 | >0.05 | -1.39 | >0.05 | -1.61 | 0.02 | -1.46 | 0.04 |
| Homeostasis | | |  |  |  |  |  |  |  |  |  |  |
| Ppargc1b | Peroxisome proliferative activated receptor, gamma, coactivator 1 beta | | -1.66 | 0.02 | -1.49 | >0.05 | -1.81 | 5.40E-04 | -1.81 | 1.06E-03 | -1.6 | 2.97E-03 |
| Lipid, fatty acid and steroid metabolism | | |  |  |  |  |  |  |  |  |  |  |
| Ppargc1b | Peroxisome proliferative activated receptor, gamma, coactivator 1 beta | | -1.66 | 0.02 | -1.49 | >0.05 | -1.81 | 5.40E-04 | -1.81 | 1.06E-03 | -1.6 | 2.97E-03 |
| Dgkg | Diacylglycerol kinase, gamma | | -1.46 | >0.05 | -1.41 | >0.05 | -1.45 | >0.05 | -1.52 | >0.05 | -1.59 | 0.02 |
| Other metabolism | | |  |  |  |  |  |  |  |  |  |  |
| Map3k1 | Mitogen-activated protein kinase kinase kinase 1 | | -1.46 | >0.05 | -1.45 | >0.05 | -1.56 | 5.60E-03 | -1.84 | 6.18E-04 | -1.65 | 7.51E-04 |
| Nucleoside, nucleotide and nucleic acid metabolism | | |  |  |  |  |  |  |  |  |  |  |
| BC039093 | cDNA sequence BC039093 | | -1.84 | 9.07E-04 | -1.54 | >0.05 | -1.7 | 2.06E-03 | -1.92 | 1.33E-04 | -1.73 | 1.73E-04 |
| Phf17 | PHD finger protein 17 | | -1.7 | 0.01 | -1.59 | 0.03 | -1.62 | 0.01 | -1.72 | 4.73E-03 | -1.55 | 8.58E-03 |
| Ppargc1b | Peroxisome proliferative activated receptor, gamma, coactivator 1 beta | | -1.66 | 0.02 | -1.49 | >0.05 | -1.81 | 5.40E-04 | -1.81 | 1.06E-03 | -1.6 | 2.97E-03 |
| Dbp | D site albumin promoter binding protein | | -1.59 | >0.05 | -1.47 | >0.05 | -1.54 | 0.04 | -1.6 | 0.03 | -1.57 | 0.01 |
| Zfp41 | Zinc finger protein 41 | | -1.53 | >0.05 | -1.4 | >0.05 | -1.54 | 0.04 | -1.58 | 0.04 | -1.54 | 0.02 |
| Tlk1 | Tousled-like kinase 1 | | -1.49 | 0.04 | -1.39 | >0.05 | -1.43 | >0.05 | -1.55 | >0.05 | -1.52 | 8.56E-03 |
| Mybl2 | Myeloblastosis oncogene-like 2 | | -1.48 | >0.05 | -1.42 | >0.05 | -1.42 | >0.05 | -1.51 | >0.05 | -1.51 | 0.02 |
| E2f2 | E2F transcription factor 2 | | -1.45 | >0.05 | -1.37 | >0.05 | -1.38 | >0.05 | -1.56 | 0.04 | -1.52 | 0.04 |

| Gene symbol | | Description | 1119-3 | | C10 | | C29 | | D6 | | D7 | |
| --- | --- | --- | --- | --- | --- | --- | --- | --- | --- | --- | --- | --- |
|  |  |  | FC | P-value | FC | P-value | FC | P-value | FC | P-value | FC | P-value |
| Nucleoside, nucleotide and nucleic acid metabolism *(Continued)* | | | | | |  |  |  |  |  |  |  |
| LOC670044 | Similar to Mothers against decapentaplegic homolog 6 (SMAD 6) (Mothers against DPP homolog 6) (Smad6) (Mad homolog 7) | | -1.44 | >0.05 | -1.35 | >0.05 | -1.48 | >0.05 | -1.57 | 0.04 | -1.45 | >0.05 |
| Phosphate metabolism | | |  |  |  |  |  |  |  |  |  |  |
| Map3k1 | Mitogen-activated protein kinase kinase kinase 1 | | -1.46 | >0.05 | -1.45 | >0.05 | -1.56 | 5.60E-03 | -1.84 | 6.18E-04 | -1.65 | 7.51E-04 |
| Biological process unclassified | | |  |  |  |  |  |  |  |  |  |  |
| Cytip | Cytohesin 1 interacting protein | | -2.9 | 1.62E-11 | -2.16 | 1.08E-06 | -2.35 | 1.19E-08 | -2.82 | 6.46E-12 | -2.53 | 3.53E-13 |
| Klhl6 | Kelch-like 6 (Drosophila) | | -2.1 | 5.71E-07 | -1.73 | 3.39E-04 | -1.73 | 8.32E-05 | -2.15 | 2.06E-06 | -1.91 | 1.06E-06 |
| Slc40a1 | Solute carrier family 40 (iron-regulated transporter), member 1 | | -1.95 | 5.21E-06 | -1.83 | 2.61E-05 | -1.8 | 1.17E-05 | -2.31 | 1.15E-07 | -1.92 | 6.60E-07 |
| Tmem86a | Transmembrane protein 86A | | -1.85 | 1.23E-03 | -1.51 | >0.05 | -1.79 | 7.58E-04 | -2.02 | 2.43E-05 | -1.79 | 4.44E-04 |
| Tmem51 | Transmembrane protein 51 | | -1.81 | 3.69E-04 | -1.57 | 0.01 | -1.65 | 8.54E-04 | -2.08 | 9.03E-06 | -1.86 | 4.84E-06 |
| Lhfpl2 | Lipoma HMGIC fusion partner-like 2 | | -1.78 | 1.60E-04 | -1.52 | 0.02 | -1.8 | 8.18E-06 | -1.96 | 6.97E-05 | -1.82 | 6.49E-06 |
| Slc37a1 | 10 days neonate skin cDNA, RIKEN full-length enriched library, clone:4732478E01 product:solute carrier family 37 (glycerol-3-phosphate transporter), member 1, full insert sequence | | -1.78 | 3.92E-03 | -1.57 | 0.04 | -1.61 | 0.02 | -1.8 | 1.16E-03 | -1.73 | 2.98E-04 |
| C130050O18Rik | RIKEN cDNA C130050O18 gene | | -1.78 | 3.88E-03 | -1.62 | 0.02 | -1.85 | 2.48E-04 | -1.94 | 1.12E-04 | -1.81 | 1.05E-04 |
| AI595366 | Leucine rich repeat containing 14B | | -1.77 | 3.95E-03 | -1.51 | >0.05 | -1.59 | 0.02 | -1.85 | 4.90E-04 | -1.63 | 0.01 |
| B930041F14Rik | RIKEN cDNA B930041F14 gene | | -1.75 | 4.00E-03 | -1.54 | >0.05 | -1.67 | 3.77E-03 | -1.87 | 3.39E-04 | -1.74 | 1.36E-04 |
| LOC100045981 | Similar to synaptotagmin XI | | -1.74 | 5.45E-03 | -1.58 | 0.04 | -1.65 | 6.70E-03 | -1.97 | 5.60E-05 | -1.85 | 1.29E-05 |
| Arrdc3 | Arrestin domain containing 3 | | -1.74 | 6.50E-03 | -1.6 | 0.03 | -1.71 | 2.72E-03 | -1.95 | 9.29E-05 | -1.55 | 0.02 |

| Gene symbol | | Description | 1119-3 | | C10 | | C29 | | D6 | | D7 | |
| --- | --- | --- | --- | --- | --- | --- | --- | --- | --- | --- | --- | --- |
|  |  |  | FC | P-value | FC | P-value | FC | P-value | FC | P-value | FC | P-value |
| Biological process unclassified (*Continued*) | | |  |  |  |  |  |  |  |  |  |  |
| Lzts2 | Leucine zipper, putative tumor suppressor 2 | | -1.72 | 8.22E-03 | -1.63 | 0.02 | -1.74 | 1.82E-03 | -1.87 | 3.22E-04 | -1.76 | 9.06E-04 |
| 1200015N20Rik | Family with sequence similarity 13, member C (Fam13c), transcript variant 1 | | -1.68 | 0.02 | -1.62 | 0.02 | -1.7 | 3.48E-03 | -1.77 | 1.78E-03 | -1.85 | 7.55E-05 |
| A630006E02Rik | 3 days neonate thymus cDNA, RIKEN full-length enriched library, clone:A630006E02 product:similar to MYELOBLAST KIAA0223 (FRAGMENT) [Homo sapiens], full insert sequence | | -1.65 | 0.02 | -1.56 | 0.04 | -1.76 | 1.30E-03 | -1.8 | 1.25E-03 | -1.48 | >0.05 |
| Fbxo21 | F-box protein 21 | | -1.62 | 0.02 | -1.4 | >0.05 | -1.57 | 0.02 | -1.67 | 0.01 | -1.53 | 0.01 |
| Plekhm1 | Pleckstrin homology domain containing, family M (with RUN domain) member 1 | | -1.61 | 6.50E-03 | -1.37 | >0.05 | -1.53 | 7.28E-03 | -1.62 | 0.02 | -1.49 | 0.02 |
| 6330416G13Rik | RIKEN cDNA 6330416G13 gene | | -1.59 | >0.05 | -1.49 | >0.05 | -1.62 | 0.01 | -1.74 | 3.44E-03 | -1.69 | 9.91E-04 |
| Hmha1 | Histocompatibility (minor) HA-1 | | -1.59 | 0.01 | -1.43 | >0.05 | -1.49 | 0.02 | -1.65 | 0.01 | -1.51 | 0.01 |
| Clspn | Claspin homolog (Xenopus laevis) | | -1.59 | 0.04 | -1.34 | >0.05 | -1.35 | >0.05 | -1.56 | 0.04 | -1.46 | 0.04 |
| LOC380927 | Similar to Poly(A) binding protein, cytoplasmic 4, isoform 1 | | -1.58 | 9.03E-03 | -1.37 | >0.05 | -1.53 | 7.09E-03 | -1.6 | 0.03 | -1.51 | 9.91E-03 |
| Pacs2 | Phosphofurin acidic cluster sorting protein 2 | | -1.57 | 0.03 | -1.37 | >0.05 | -1.42 | >0.05 | -1.52 | >0.05 | -1.47 | 0.03 |
| Fam13c | Family with sequence similarity 13, member C | | -1.57 | >0.05 | -1.48 | >0.05 | -1.53 | >0.05 | -1.72 | 4.89E-03 | -1.67 | 3.40E-03 |
| Tgfbr1 | Transforming growth factor, beta receptor I | | -1.55 | 0.03 | -1.44 | >0.05 | -1.47 | 0.03 | -1.61 | 0.02 | -1.62 | 1.48E-03 |
| LOC100045343 | Similar to CDNA sequence BC046404 | | -1.55 | 0.02 | -1.39 | >0.05 | -1.47 | 0.02 | -1.58 | 0.04 | -1.56 | 3.14E-03 |
| A130092J06Rik | Family with sequence similarity 78, member A | | -1.54 | >0.05 | -1.35 | >0.05 | -1.59 | 9.39E-03 | -1.73 | 4.16E-03 | -1.66 | 8.43E-04 |
| Klhl21 | Kelch-like 21 (Drosophila) | | -1.53 | >0.05 | -1.37 | >0.05 | -1.52 | >0.05 | -1.57 | 0.04 | -1.47 | 0.04 |

| Gene symbol | | Description | 1119-3 | | C10 | | C29 | | D6 | | D7 | |
| --- | --- | --- | --- | --- | --- | --- | --- | --- | --- | --- | --- | --- |
|  |  |  | FC | P-value | FC | P-value | FC | P-value | FC | P-value | FC | P-value |
| Biological process unclassified (*Continued*) | | |  |  |  |  |  |  |  |  |  |  |
| Cep78 | Centrosomal protein 78 | | -1.51 | >0.05 | -1.43 | >0.05 | -1.48 | 0.03 | -1.58 | 0.04 | -1.51 | 0.02 |
| Wipf1 | WAS/WASL interacting protein family, member 1 | | -1.49 | >0.05 | -1.36 | >0.05 | -1.29 | >0.05 | -1.63 | 0.02 | -1.44 | >0.05 |
| Ypel3 | Yippee-like 3 (Drosophila) | | -1.49 | >0.05 | -1.4 | >0.05 | -1.43 | >0.05 | -1.55 | >0.05 | -1.52 | 0.02 |
| 2310043N10Rik | Nuclear paraspeckle assembly transcript 1 (non-protein coding) (Neat1) | | -1.49 | >0.05 | -1.6 | 0.03 | -1.76 | 1.19E-03 | -1.59 | 0.04 | -1.46 | 0.04 |
| Snx24 | Sorting nexing 24 | | -1.49 | >0.05 | -1.35 | >0.05 | -1.39 | >0.05 | -1.64 | 0.02 | -1.46 | 0.03 |
| 1810011H11Rik | RIKEN cDNA 1810011H11 gene | | -1.46 | >0.05 | -1.34 | >0.05 | -1.39 | >0.05 | -1.6 | 0.03 | -1.46 | 0.04 |
| scl0015365.1_6 | High mobility group AT-hook 2, pseudogene 1, mRNA (cDNA clone IMAGE:1195052), partial cds | | -1.46 | >0.05 | -1.33 | >0.05 | -1.64 | 4.80E-03 | -1.66 | 0.01 | -1.49 | 0.02 |
| 6430548M08Rik | RIKEN cDNA 6430548M08 gene, transcript variant 1 | | -1.43 | >0.05 | -1.39 | >0.05 | -1.43 | >0.05 | -1.58 | 0.04 | -1.45 | >0.05 |
| Casp2 | 13 days embryo lung cDNA, RIKEN full-length enriched library, clone:D430002B11 product:caspase 2, full insert sequence | | -1.42 | >0.05 | -1.39 | >0.05 | -1.47 | 0.04 | -1.61 | 0.03 | -1.47 | 0.03 |
| Snhg10 | Small nucleolar RNA host gene (non-protein coding) 10 | | -1.41 | >0.05 | -1.45 | >0.05 | -1.63 | 0.01 | -1.57 | 0.04 | -1.54 | 0.01 |
| EG433224 | Predicted gene 5512 (Gm5512) | | -1.41 | >0.05 | -1.37 | >0.05 | -1.42 | >0.05 | -1.49 | >0.05 | -1.5 | 0.02 |
| Dennd3 | DENN/MADD domain containing 3 | | -1.39 | >0.05 | -1.32 | >0.05 | -1.38 | >0.05 | -1.51 | >0.05 | -1.55 | 0.02 |
